# RNA-Seq Transcriptome Analysis of Peripheral Blood From Cattle Infected With *Mycobacterium bovis* Across an Experimental Time Course

**DOI:** 10.3389/fvets.2021.662002

**Published:** 2021-05-28

**Authors:** Kirsten E. McLoughlin, Carolina N. Correia, John A. Browne, David A. Magee, Nicolas C. Nalpas, Kevin Rue-Albrecht, Adam O. Whelan, Bernardo Villarreal-Ramos, H. Martin Vordermeier, Eamonn Gormley, Stephen V. Gordon, David E. MacHugh

**Affiliations:** ^1^Animal Genomics Laboratory, UCD School of Agriculture and Food Science, UCD College of Health and Agricultural Sciences, University College Dublin, Dublin, Ireland; ^2^TB Immunology and Vaccinology Team, Department of Bacteriology, Animal and Plant Health Agency, Weybridge, United Kingdom; ^3^UCD School of Veterinary Medicine, UCD College of Health and Agricultural Sciences, University College Dublin, Dublin, Ireland; ^4^UCD Conway Institute of Biomolecular and Biomedical Research, University College Dublin, Dublin, Ireland

**Keywords:** biomarker, cattle, gene expression, host-pathogen interaction, immune response, time series, tuberculosis, *Mycobacterium bovis*

## Abstract

Bovine tuberculosis, caused by infection with members of the *Mycobacterium tuberculosis* complex, particularly *Mycobacterium bovis*, is a major endemic disease affecting cattle populations worldwide, despite the implementation of stringent surveillance and control programs in many countries. The development of high-throughput functional genomics technologies, including RNA sequencing, has enabled detailed analysis of the host transcriptome to *M. bovis* infection, particularly at the macrophage and peripheral blood level. In the present study, we have analysed the transcriptome of bovine whole peripheral blood samples collected at −1 week pre-infection and +1, +2, +6, +10, and +12 weeks post-infection time points. Differentially expressed genes were catalogued and evaluated at each post-infection time point relative to the −1 week pre-infection time point and used for the identification of putative candidate host transcriptional biomarkers for *M. bovis* infection. Differentially expressed gene sets were also used for examination of cellular pathways associated with the host response to *M. bovis* infection, construction of *de novo* gene interaction networks enriched for host differentially expressed genes, and time-series analyses to identify functionally important groups of genes displaying similar patterns of expression across the infection time course. A notable outcome of these analyses was identification of a 19-gene transcriptional biosignature of infection consisting of genes increased in expression across the time course from +1 week to +12 weeks post-infection.

## Introduction

Bovine tuberculosis (BTB) is caused by *Mycobacterium bovis* and other intracellular bacterial pathogens of the *Mycobacterium tuberculosis* complex (MTBC), which display 99.9% DNA sequence identity at the genome level (1–3). Each member of the MTBC has a distinctive host spectrum, such that tuberculosis (TB) affects a wide range of mammals including humans (4). In addition, BTB has been classified as the fourth most important disease of livestock in terms of zoonotic and economic impact globally (5, 6). It has also been conservatively estimated that BTB costs $3 billion annually and imposes a large financial burden on farmers with infected herds (7, 8). Furthermore, as a zoonosis, *M. bovis* infection has important implications for human health; transmission from cattle to humans does occur and is responsible for a small but significant number of human TB cases, particularly in developing countries (9–11).

Tuberculous mycobacteria—primarily *M. bovis* and *M. tuberculosis*, the main cause of human TB—are generally inhaled from the environment within aerosol droplets and are phagocytosed by host alveolar macrophages (AMs); therefore, infection is normally initiated within, and restricted to, lung tissues (12–15). Tuberculous mycobacteria have evolved a wide range of mechanisms to modulate, suppress, and manipulate specific host immune mechanisms, including inhibition of phagosomal maturation, detoxification of reactive oxygen and nitrogen species (ROS and RNS), repair of ROS- and RNS-induced cellular damage, resistance to antimicrobial and cytokine defences, modulation of antigen presentation, and induction of cellular necrosis and inhibition of apoptosis (16–19). Tuberculous disease is characterised by lesions located at the site of infection, which are formed when AMs and other immune cells engage and eliminate most of the bacilli. The remaining intact mycobacterial cells are confined in granulomas that act to contain the infection, but may, under certain conditions, actually facilitate expansion and dissemination of mycobacteria to spread infection (20–22).

In Ireland, a test and slaughter policy for BTB was introduced in the early 1950s as part of the national BTB eradication scheme (23, 24). This policy includes compulsory screening of all animals using the single intradermal comparative tuberculin test alone or in conjunction with an *in vitro* enzyme-linked immunosorbent assay–based interferon γ (IFN-γ) release assay (IGRA) that increases the sensitivity of diagnosis (25). However, limitations of current diagnostic methods prevent early and accurate detection and subsequent removal of all infected animals from a herd, thereby contributing to the ongoing persistence of BTB, which continues to impact cattle production in Ireland, the United Kingdom, and other countries (24, 26). Therefore, the most important objective of an effective BTB control strategy—to identify and remove all infected cattle from a herd regardless of the stage of infection—is substantially hindered by current diagnostic technologies. Novel methods of BTB diagnosis are urgently required to augment current test procedures in conjunction with appropriate wildlife reservoir control measures (27).

In recent years, the availability of a well-annotated bovine genome sequence combined with high-throughput functional genomics technologies has provided an unprecedented opportunity to gain a deeper understanding of host–pathogen interaction, identify blood-derived RNA-based biomarkers, and develop new diagnostic methods for BTB caused by infection with *M. bovis* (28–33).

Previous transcriptomics studies of the host immune response to *M. bovis* have been performed using blood-derived RNA obtained from both naturally and experimentally infected animals, as it has been shown that host immune responses occurring in peripheral blood reflect those at the primary site of disease in BTB (34). In this regard, the dynamic transcriptome of circulating blood, which contains a large pool of “biosensors” in the form of RNA transcripts, can reflect physiological and pathological events occurring elsewhere in different tissues and organs, thereby providing a comprehensive overview of the status of the immune system (35, 36). In addition, peripheral blood has provided information on the pathobiology of many diseases; it is accessible and easily collected, making it ideally suited for the development of diagnostic biomarker tests based on transcriptional profiling (37–39).

For the experimental work described here, RNA sequencing (RNA-seq) was used to study the bovine whole peripheral blood transcriptome in response to infection with *M. bovis* across a large-scale 14-week animal infection time course. The main objectives of the study were to examine the host peripheral blood transcriptional responses across the early stages of *M. bovis* infection and identify differentially expressed (DE) genes across the infection time course that represent promising candidate biomarkers for BTB. In addition, we aimed to identify host canonical pathways and interaction networks enriched for DE genes, which may shed light on the immunobiology of *M. bovis* infection in cattle. We also used time-series analysis and Gene Ontology (GO) information to identify functionally important groups of DE genes across the infection time course.

## Materials and Methods

### Overview of Animal Infection Time Course Experiment

**Figure 1:**
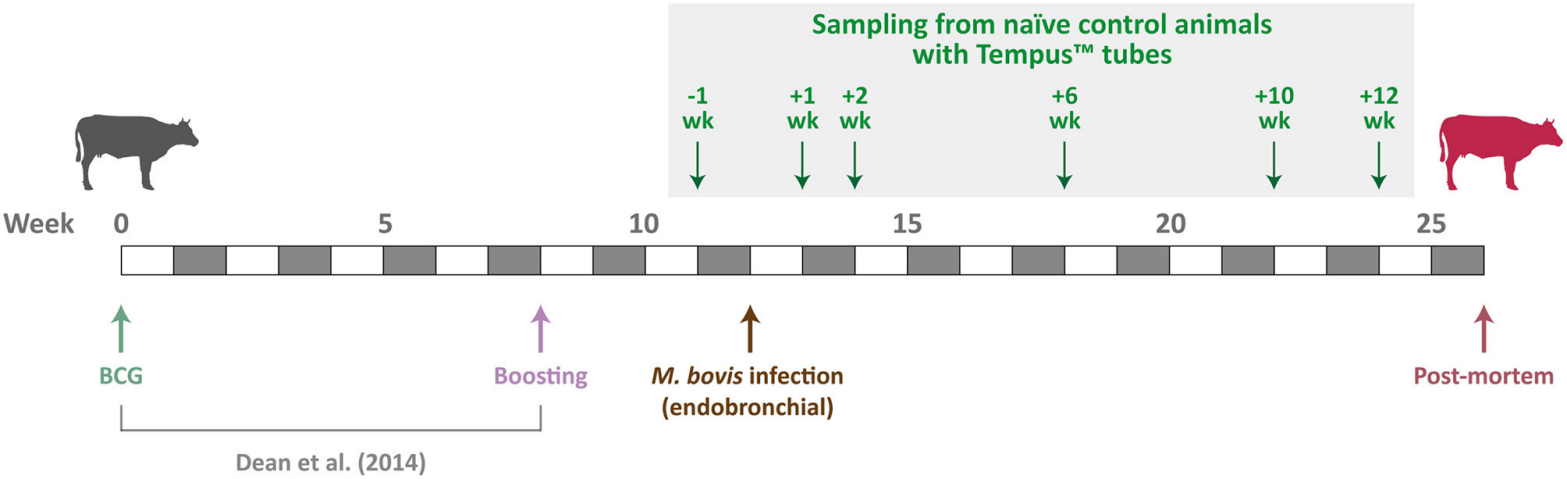
Schedule for the *M. bovis* infection time-course experiment. Sampling time points for the 10 non-vaccinated control cattle used are indicated by green arrows.

Animal resources for the present study were obtained from a 26-week vaccination and challenge experiment of age- and sex-matched cattle infected with *M. bovis* (40–44). Ten male age-matched Holstein–Friesian calves (4–6 months old) were sourced from farms known to be free of BTB disease. The animals used for the experimental work described here were the naive control group (non-vaccinated) for a vaccine efficacy study (40). Figure 1 shows the experimental schedule used by Dean et al. (40) and details the sampling time points for the 10 non-vaccinated control cattle used for the research work described here.

### Inoculation With *M. bovis* Strain AF2122/97

The challenge strain, *M. bovis* AF2122/97 (2, 45), was delivered endobronchially at 2 × 10^3^ colony-forming units per animal using the following procedure described by Whelan et al. (46). Prior to endobronchial inoculation animals were sedated with Rompun® (Bayer Animal Health, Newbury, UK) according to the manufacturer's instructions. Following this, an LSVP 22 VGS89x14 endoscope (Veterinary Endoscopy Services, Welshpool, UK) lubricated with Vet-Lubigel (Millpledge Veterinary, Clarborough, UK) was inserted through a nostril into the trachea and placed above the bronchial opening to the cardiac lobe and the main bifurcation between left and right lobes. A cannula of 1.8-mm internal diameter (Veterinary Endoscopy Services) was inserted through the endoscope and used to deliver the *M. bovis* AF2122/97 inoculum in 2 mL of phosphate-buffered saline (PBS). Following this, an additional 2 mL of PBS was then used to wash the cannula, and the cannula and endoscope were withdrawn. For each individual animal, a new sterile cannula was used, the internal channel of the endoscope, through which the cannula was inserted, was rinsed with 20 mL of PBS, and the outside of the endoscope was cleaned thoroughly with sterilising tissue wipes (Medichem International, Sevenoaks, UK).

Individual responses to infection across the time course and disease pathology for the animals used in this study have been described in detail previously and include whole-blood IFN-γ assay, evaluation of peripheral blood mononuclear cell (PBMC) cytokine responses by intracellular cytokine staining, gross (visible) pathology and histopathology, and evaluation of bacterial load in lymph nodes (40, 41).

### Peripheral Blood Collection and Total RNA Extraction

Approximately 3 mL of *ex vivo* peripheral blood was sampled from all 10 naive control animals at −1 week pre-infection and then at +1, +2, +6, +10, and +12 weeks post-infection (Figure 1). All blood samples were obtained during the morning (between 7:00 and 10:00 a.m.) of each collection day and directly collected into Tempus™ blood RNA tubes (Applied Biosystems®/Thermo Fisher Scientific, Warrington, UK). Immediately after blood collection at each time point, Tempus™ tube samples for each animal were vortexed for ~10 s to ensure complete red blood cell lysis. Tempus™ tube blood lysate samples for animals at each of the nine time points were then stored at −80°C until they were used for total RNA extraction and purification.

The Tempus™ Spin RNA Isolation Kit (Applied Biosystems®/Thermo Fisher Scientific) was used for total RNA extraction and purification using the following protocol provided by the manufacturer. Tempus™ tube blood lysate samples were thawed at room temperature prior to RNA extraction and purification. Once thawed, for each sample, ~3 mL of blood lysate was transferred to a 50-mL plastic centrifuge tube, and PBS was added to a final volume of 12 mL. Each sample was then mixed by vortexing for 30 s and then centrifuged at 3,000 × *g* for 30 min at 4°C. The supernatant was then removed, and the remaining RNA-containing pellet was resuspended with a brief vortex in 400 μL of the proprietary RNA Purification Resuspension Solution. Following this, the resuspended RNA sample was pipetted into the RNA purification filter inserted into a 1.5-mL microcentrifuge tube for waste collection. The RNA purification filter/microcentrifuge tube was then centrifuged at 16,000 × *g* for 30 s, and the liquid waste and microcentrifuge tube discarded. The RNA purification filter was then placed in a clean microcentrifuge tube, 500 μL of proprietary RNA Purification Wash Solution 1 was added, followed by another centrifugation step at 16,000 × *g* for 30 s and disposal of the liquid waste and microcentrifuge tube. This step was then repeated using 500 μL of proprietary RNA Purification Wash Solution 2 with a centrifugation step at 16,000 × *g* for 30 s. A final wash step was then performed with 500 μL of RNA Purification Wash Solution 2 and centrifugation at 16,000 × *g* for 30 s followed by disposal of the liquid waste and microcentrifuge tube. The RNA purification filter was then placed in a clean microcentrifuge tube and centrifuged at 16,000 × *g* for 30 s to dry the membrane. The RNA purification filter was then inserted into a clean RNase-free collection microcentrifuge tube and 100 μL of Nucleic Acid Purification Elution Solution was added and incubated for 2 min followed by centrifugation at 16,000 × *g* for 30 s; the RNA eluate was then pipetted back onto the filter membrane, and the centrifugation step was repeated. Approximately 90 μL of the final RNA eluate was then pipetted (avoiding particulate material) into a new labelled RNase-free collection microcentrifuge for long-term storage at −80°C.

### RNA Quality Checking and Quantification

RNA quantity and quality checking were performed using a NanoDrop™ 1000 spectrophotometer (Thermo Fisher Scientific, Waltham, MA, USA) and an Agilent 2100 Bioanalyzer using an RNA 6000 Nano LabChip kit (Agilent Technologies, Cork, Ireland). The majority of samples displayed a 260/280 ratio >1.8 and RNA integrity numbers >8.0 (Supplementary Table 1 in Supplementary Material 1). RNA quality and quantity checking revealed that three samples did not have measurable quantities of RNA, and these were excluded from downstream RNA-seq library preparation (15, ID 6520, +2 weeks; 21, ID 6522, +2 weeks; and 27, ID 6526, + 2 weeks).

### Strand-Specific RNA-Seq Library Preparation and Sequencing

For RNA-seq library preparation, 1 μg of total RNA from each sample was used to prepare individually barcoded strand-specific RNA-seq libraries. Two rounds of poly(A)^+^ RNA purification were performed for all RNA samples using the Dynabeads® mRNA DIRECT™ Micro Kit (Ambion®/Thermo Fisher Scientific, Austin, TX, USA) according to the manufacturer's instructions. The purified poly(A)^+^ RNA was then used to generate strand-specific RNA-seq libraries using the ScriptSeq™ v2 RNA-Seq Library Preparation Kit, the ScriptSeq™ Index PCR Primers (sets 1–4), and the FailSafe™ PCR enzyme system (all sourced from Epicentre®/Illumina® Inc., Madison, WI, USA) according to the manufacturer's instructions. RNA-seq libraries were purified using the Agencourt® AMPure® XP system (Beckman Coulter Genomics, Danvers, MA, USA) according to the manufacturer's instructions for double size selection (0.75 × followed by 1.0 × ratio). RNA-seq libraries were quantified using a Qubit® fluorometer and Qubit® dsDNA HS Assay Kit (Invitrogen™/Thermo Fisher Scientific, Carlsbad, CA, USA), whereas library quality cheques were performed using an Agilent 2100 Bioanalyzer and High Sensitivity DNA Kit (Agilent Technologies Ltd.). Individually barcoded RNA-seq libraries were pooled in equimolar quantities, and the quantity and quality of the final pooled libraries (three pools in total) were assessed as described previously. RNA-seq library sample barcode index sequences are detailed in Supplementary Table 1 (Supplementary Material 1).

Prior to high-throughput sequencing, the content of several RNA-seq libraries was validated using conventional Sanger dideoxy sequencing. Library inserts from 16 libraries were cloned using the Zero Blunt® TOPO® PCR Cloning Kit according to the manufacturer's instructions (Invitrogen™/Thermo Fisher Scientific). Sanger sequencing of 36 plasmid inserts from these selected libraries confirmed that the RNA-seq libraries contained inserts derived from bovine mRNA. Plasmid sequencing was outsourced (Source Bioscience Ltd., Dublin, Ireland), and sequences generated were validated using BLAST searching of the DNA sequence database (47). Cluster generation and high-throughput sequencing of the pooled RNA-seq libraries were performed using an Illumina® HiSeq™ 2000 Sequencing System at the MSU Research Technology Support Facility (RTSF) Genomics Core (https://rtsf.natsci.msu.edu/genomics; Michigan State University, MI, USA). Each of the three pooled libraries was sequenced independently on five lanes split across multiple Illumina® flow cells. The pooled libraries were sequenced as paired-end 2 × 100 nucleotide reads using Illumina® version 5.0 sequencing kits. Additionally, after exploratory data analysis (Supplementary Figures 1, 2), it was decided to remove animal ID 6522 completely from the analysis and proceed with 52 RNA-seq sample data sets (Supplementary Table 1 in Supplementary Material 1). All RNA-seq data generated for this study have been deposited in the European Nucleotide database with experiment series accession numbers (PRJEB27764 and PRJEB44470).

### Bioinformatics Analyses of RNA-Seq Data

Except where indicated, bioinformatics procedures and analyses were performed on a 32-core Compute Server running Linux Ubuntu (version 12.04.2) hosted at the UCD Research IT Data Centre (stampede.ucd.ie) and administered by the UCD Animal Genomics Group. All of the bioinformatics workflow/pipeline components including Linux Bash, Perl, and R scripts used were deposited in a GitHub repository (https://github.com/kmcloughlin1/RNA-sequencing) and were modified from published methods described by our group (48). Supplementary Figure 3 shows a schematic of the complete RNA-seq bioinformatics workflow and the downstream tools used for time-series analysis and various systems biology methods.

Deconvolution (filtering and segregation of sequence reads based on the unique RNA-seq library barcode index sequences; Supplementary Table 1 in Supplementary Material 1) was performed by the MSU RTSF Genomics Core using a pipeline that simultaneously demultiplexed and converted pooled sequence reads to discrete FASTQ files for each RNA-seq sample with no barcode index mismatches permitted. The RNA-seq FASTQ sequence read data were then downloaded from the MSU RTSF Genomics Core FTP server, and a custom Perl script was used to filter out paired-end reads containing adapter sequence contamination (with up to three mismatches allowed) and to remove poor quality paired-end reads (i.e., one or both reads containing 25% of bases with a Phred quality score <20). The quality of individual RNA-seq sample library files was then reassessed postfiltering using the FastQC software package (version 0.10.1) (49).

Paired-end reads, from each filtered individual library, were aligned to the *Bos taurus* reference genome (UMD3.1.73) (50) using the STAR aligner software package (version 2.3.0) (51). For each library, raw counts for each gene based on the sense strand data were obtained using the featureCounts software from the Subread package (version 1.3.5-p4) (52). The featureCounts parameters were set to unambiguously assign uniquely aligned paired-end reads in a stranded manner to the exons of genes within the UMD3.1.73 *B. taurus* reference genome annotation. The gene count outputs were then used to perform differential gene expression analysis using the edgeR Bioconductor package (version 3.2.4) (53) within an R-based pipeline that was customised to perform the following functions:

1. Use biomaRt (54) to generate a detailed bovine gene annotation for downstream analyses, then filter out all bovine rRNA genes.
2. Filter out genes displaying expression levels below a minimal detection threshold of one count per million in at least *n* = 9 individual libraries (where n = smallest group of biological replicates).
3. Calculate normalisation factors for each library using the trimmed mean of M values method (55).
4. Identify DE genes between the pre-infection animal group (−1 week) and each of the post-infection animal groups (+1, +2, +6, +10, and +12 weeks) using a paired-sample approach with the edgeR package. Differential expression was evaluated by fitting a negative binomial generalised linear model for each gene.
5. Correct for multiple testing using the Benjamini–Hochberg method (56) with a false discovery rate (FDR) threshold of ≤ 0.05.

### Systems Analyses of RNA-Seq Gene Expression Data

The Ingenuity® Pathway Analysis (IPA) software package (57) with the Ingenuity® Knowledge Base (Qiagen Corp., Redwood City, CA, USA; release date July 2014; www.ingenuity.com) was used to identify overrepresented (enriched) canonical pathways and construction of biological interaction networks for sets of DE genes at each post-infection time point (+1, +2, +6, +10, and +12 weeks) compared to the pre-infection time point (−1 week). For identification of overrepresented canonical pathways, a multiple testing correction (Benjamini–Hochberg method) was applied with an FDR threshold ≤ 0.05. The IPA Biomarker Filter tool was also used to identify and prioritise molecular biomarker candidates such that only experimentally observed and high-confidence predicted biological relationships were included. All the IPA data sources were used for three mammalian species in the IPA Knowledge Base (*Homo sapiens, Mus musculus, Rattus norvegicus*). Biological interaction networks were ranked according to Network Score values generated algorithmically by IPA and based on the hypergeometric distribution and calculated with the right-tailed Fisher exact test (58).

### Time-Series Analysis of RNA-Seq Gene Expression Data

Time-series analysis of gene expression data from the animal infection time-course experiment was performed using the Short Time-series Expression Miner (STEM) software package (59). The computational procedure for selecting model profiles that are representative and distinct is described by Ernst et al. (60). The software package implements a method for clustering short time-series expression data that can differentiate between real and random patterns of temporal gene expression changes and assigns each gene to the model profile that most closely matches the temporal gene expression profile for that gene as determined by the correlation coefficient. A permutation test is then used to determine which model profiles have a statistically significant number of genes assigned compared to random expectations from the mean number assigned to each profile based on the permuted data (59). STEM also incorporates GO enrichment functionality for biological interpretation of time-series gene expression data.

## Results and Discussion

### RNA-Seq Summary Statistics

Deconvolution and filtering of sequence reads to remove adaptor-dimer contamination yielded a mean of 20.6 ± 2.0 million reads per individual barcoded RNA-seq sample library (*n* = 52 libraries and ± SD); this corresponded to a mean of 82% reads that passed this filtering step. These filtered reads were then aligned to the *B. taurus* UMD3.1.73 genome build. This yielded a mean of 15.4 ± 1.7 million filtered reads (91.5%) that uniquely mapped to this bovine genome build with a mean mapped length of 195.6 ± 0.6 bp; a mean of 0.77 ± 0.19 million reads (4.6%) that mapped to multiple genomic locations and 0.67 ± 0.17 million reads (3.9%) that did not map to any genomic location. Further analysis demonstrated that a mean of 63.1% of the filtered uniquely mapping reads (9.7 ± 1.1 million reads) were assigned to Ensembl gene IDs for the UMD3.1.73 genome build and 36.9% (5.7 ± 0.86 million reads) were ambiguous or could not be assigned to an annotated genomic region. Supplementary Table 2 (Supplementary Material 1) and Supplementary Figure 4 show the RNA-seq summary statistics. Filtering of the RNA-seq data using 52 samples (60 minus the three technical dropouts and the animal ID 6522 samples at −1, +1, +6, +10, and +12 weeks) produced 12,406 genes suitable for downstream differential expression analysis.

Multidimensional scaling plots (Supplementary Figure 5) demonstrated that it was not possible to differentiate the infected animals from non-infected animals during the early stages of the animal infection time course (+1 week post-infection vs. −1 week pre-infection, and +2 weeks vs. −1 week pre-infection). Conversely, discrimination of infected and non-infected animals was partially observed at +6 weeks post-infection and was clearly evident at +10 weeks post-infection, but this pattern of discrimination effectively disappeared by +12 weeks post-infection. Previous work by our group has shown that microarray and RNA-seq gene expression data sets from peripheral blood leukocytes (PBLs) can be used to unambiguously discriminate *M. bovis*–infected from non-infected control cattle (28, 31). However, it is important to note that the *M. bovis*–infected animals used for these earlier studies were heavily infected animals, which were maintained for ongoing disease surveillance and potency testing of diagnostic reagents.

### Differentiation of *M. bovis*–Infected and Non-infected Control Groups: Toward the Development of Transcriptomics-Based Biomarkers

The concept and the implications regarding biomarker identification and biosignature development for infectious disease have been explored thoroughly by Chaussabel et al. (36, 61, 62). In particular, these researchers emphasise that leukocytes present in peripheral blood convey valuable information about the status of the immune system that can be translated to biomarkers and onward to sensitive and specific biosignatures of infection. In addition, peripheral blood is easily accessible and can be stabilised and processed for high-throughput transcriptomics analyses using RNA-seq and other massively parallel gene expression technologies such as microarrays. It is also notable that development of transcriptional biomarkers in cattle is relatively straightforward because very low levels of globin gene transcripts (*HBA* and *HBB*) are observed in bovine peripheral blood compared to other mammalian species (63, 64).

**Figure 2:**
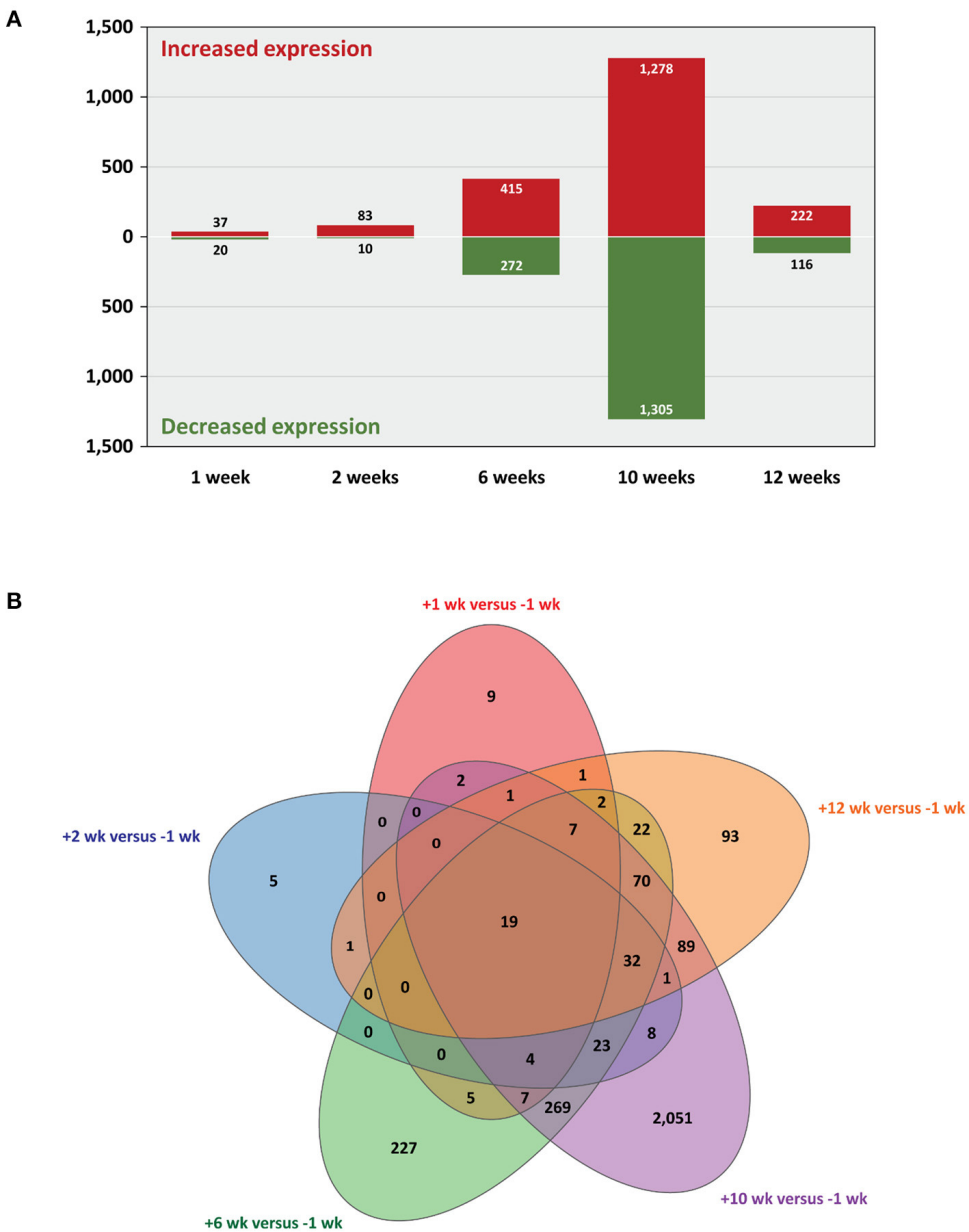
Statistically significant differentially expressed genes. Five post-infection time points are shown relative to the −1 week pre-infection time point (B-H FDR adjusted *P* ≤ 0.05). **(A)** Bar graph showing numbers of genes with increased and decreased expression and **(B)** Venn diagram showing the overlaps of DE genes for every multiple-time-point comparison.

**Figure 3:**
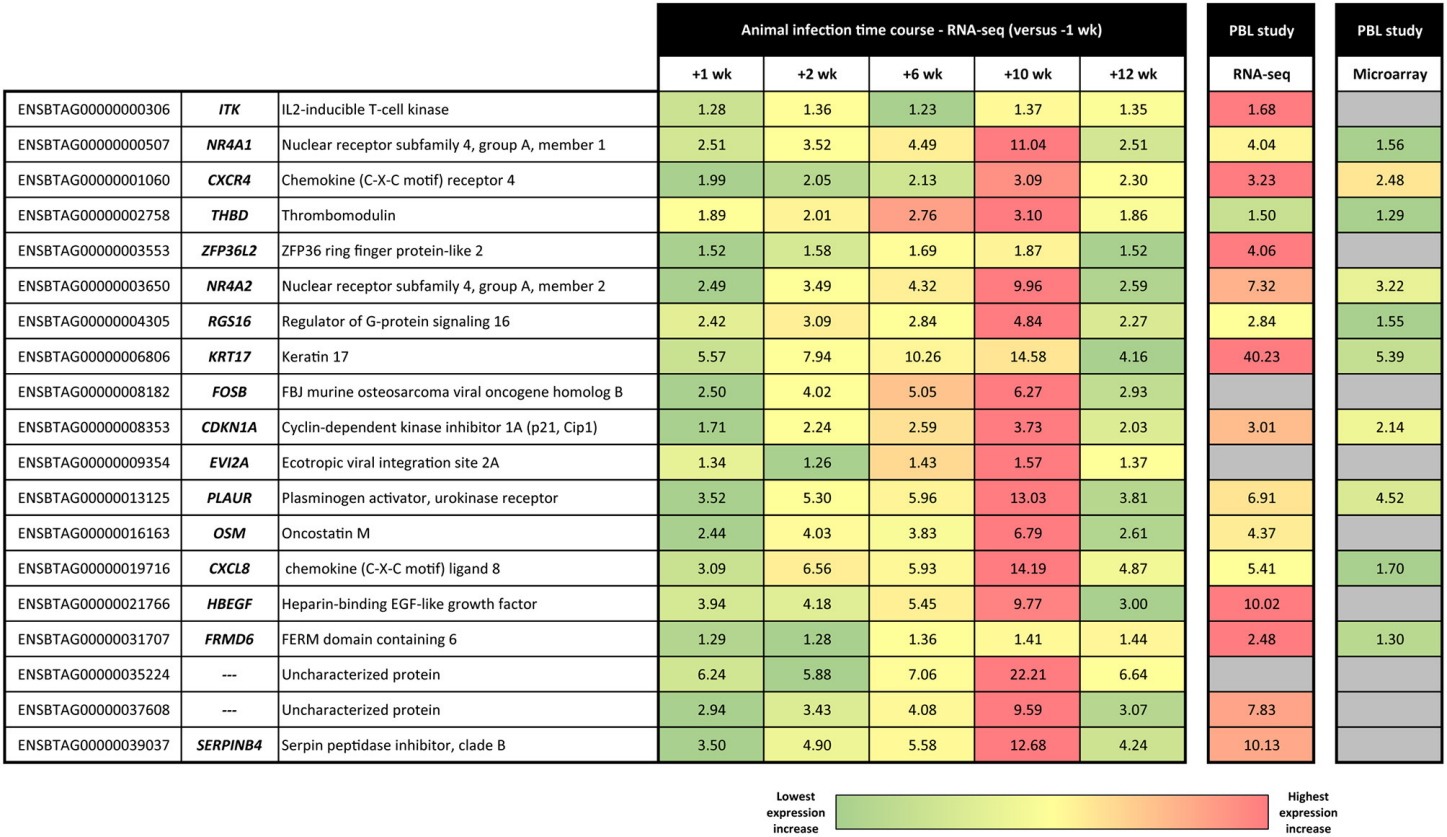
Heat map showing linear fold-change values for the panel of 19 consistently DE genes across the *M. bovis* infection course. Linear fold-change values for the post-infection time points are shown relative to the −1 week pre-infection time point. Also shown are the equivalent results obtained using RNA-seq and Affymetrix® GeneChip® Bovine Genome Array technologies by McLoughlin et al. (31).

Statistical analysis of the RNA-seq gene expression data with a B-H FDR adjusted *P* ≤ 0.05 demonstrated that differential gene expression was evident between each of the five post-infection time points (+1, +2, +6, +10, and +12 weeks) and the −1 week pre-infection time point (Figure 2A). Relatively small numbers of DE genes were detected at +1 week (37 exhibited increased and 20 exhibited decreased expression) and +2 weeks (83 increased and 10 decreased); however, the numbers of DE genes were substantially greater at +6 weeks (415 increased and 272 decreased), +10 weeks (1,278 increased and 1,305 decreased), and +12 weeks (222 increased and 116 decreased). Supplementary Tables 3–7 (Supplementary Material 2) provide detailed information on the differential expression analysis results at each of the five post-infection time points relative to the −1 week pre-infection time point for the complete set of 12,406 filtered genes. Figure 2B also shows a Venn diagram for the significant DE genes at each of the five post-infection time points (relative to the −1 week pre-infection control time point). In addition, Table 1 provides detailed information for 19 genes shown in Figure 3 that were significantly DE across all the five post-infection time points compared to the −1 week pre-infection control time point.

**Table 1:** Nineteen genes that exhibited statistically significant differential expression for each of the five post-infection time points vs. the −1 week pre-infection control time point.

**Ensembl ID**	**Gene symbol**	**Gene name**	**+1 week post-infection**		**+2 weeks post-infection**		**+6 weeks post-infection**		**+10 weeks post-infection**		**+12 weeks post-infection**	
			**Fold-change**	**B-H FDR *P*-value**	**Fold-change**	**B-H FDR *P*-value**	**Fold-change**	**B-H FDR *P*-value**	**Fold-change**	**B-H FDR *P*-value**	**Fold-change**	**B-H FDR *P*-value**
ENSBTAG00000000306	*ITK*	IL-2–inducible T-cell kinase	+1.28	0.006848	+1.36	0.000235	+1.23	0.009551	+1.37	0.000001	+1.35	0.000044
ENSBTAG00000000507	*NR4A1*	Nuclear receptor subfamily 4, group A, member 1	+2.51	0.026721	+3.52	0.000214	+4.49	0.000000	+11.04	0.000000	+2.51	0.007555
ENSBTAG00000001060	*CXCR4*	Chemokine (C-X-C motif) receptor 4	+1.99	0.004449	+2.05	0.003150	+2.13	0.000126	+3.09	0.000000	+2.30	0.000037
ENSBTAG00000002758	*THBD*	Thrombomodulin	+1.89	0.033131	+2.01	0.015038	+2.76	0.000001	+3.10	0.000000	+1.86	0.012975
ENSBTAG00000003553	*ZFP36L2*	ZFP36 ring finger protein-like 2	+1.52	0.035631	+1.58	0.020932	+1.69	0.000259	+1.87	0.000001	+1.52	0.011716
ENSBTAG00000003650	*NR4A2*	Nuclear receptor subfamily 4, group A, member 2	+2.49	0.029283	+3.49	0.000308	+4.32	0.000001	+9.96	0.000000	+2.59	0.006347
ENSBTAG00000004305	*RGS16*	Regulator of G-protein signalling 16	+2.42	0.037004	+3.09	0.000829	+2.84	0.000682	+4.84	0.000000	+2.27	0.028004
ENSBTAG00000006806	*KRT17*	Keratin 17	+5.57	0.003229	+7.94	0.000098	+10.26	0.000001	+14.58	0.000000	+4.16	0.010868
ENSBTAG00000008182	*FOSB*	FBJ murine osteosarcoma viral oncogene homologue B	+2.50	0.004449	+4.02	0.000001	+5.05	0.000000	+6.27	0.000000	+2.93	0.000079
ENSBTAG00000008353	*CDKN1A*	Cyclin-dependent kinase inhibitor 1A (p21, Cip1)	+1.71	0.049537	+2.24	0.000142	+2.59	0.000000	+3.73	0.000000	+2.03	0.000391
ENSBTAG00000009354	*EVI2A*	Ecotropic viral integration site 2A	+1.34	0.002116	+1.26	0.047028	+1.43	0.000003	+1.57	0.000000	+1.37	0.000092
ENSBTAG00000013125	*PLAUR*	Plasminogen activator, urokinase receptor	+3.52	0.001379	+5.30	0.000001	+5.96	0.000000	+13.03	0.000000	+3.81	0.000049
ENSBTAG00000016163	*OSM*	Oncostatin M	+2.44	0.029283	+4.03	0.000023	+3.83	0.000003	+6.79	0.000000	+2.61	0.004204
ENSBTAG00000019716	*CXCL8*	Chemokine (C-X-C motif) ligand 8	+3.09	0.001379	+6.56	0.000000	+5.93	0.000000	+14.19	0.000000	+4.87	0.000000
ENSBTAG00000021766	*HBEGF*	Heparin-binding EGF-like growth factor	+3.94	0.001379	+4.18	0.000308	+5.45	0.000001	+9.77	0.000000	+3.00	0.007067
ENSBTAG00000031707	*FRMD6*	FERM domain containing 6	+1.29	0.013896	+1.28	0.022194	+1.36	0.000059	+1.41	0.000001	+1.44	0.000001
ENSBTAG00000035224	—	Uncharacterized protein	+6.24	0.002116	+5.88	0.001857	+7.06	0.000071	+22.21	0.000000	+6.64	0.000304
ENSBTAG00000037608	—	Uncharacterized protein	+2.94	0.030981	+3.43	0.005467	+4.08	0.000066	+9.59	0.000000	+3.07	0.006033
ENSBTAG00000039037	*SERPINB4*	Serpin peptidase inhibitor, clade B	+3.50	0.017337	+4.90	0.000308	+5.58	0.000006	+12.68	0.000000	+4.24	0.000868

*Linear mean fold-change values are shown for each gene at each post-infection time point vs. the −1 week pre-infection control time point*.

As shown in Figure 3, there is a striking concordance between the patterns of expression for these 19 genes across the infection time course and earlier PBL microarray and RNA-seq studies published by our group (28, 31). These results provide good support for the hypothesis that a biosignature of *M. bovis* infection can be generated using transcriptomics data from cattle with early- and later-stage BTB. It also provides evidence that putative transcriptional biomarkers, identified using an experimental challenge with a relatively high *M. bovis* infectious dose, can be translated as diagnostic tools for use in naturally infected animals. Diagnostic biosignature development focusing on smaller panels of transcriptomics-based biomarkers has been used with notable success for human TB. In this case, research work has focused on specificity and differentiating active TB from latent TB and also TB disease from non-infected controls and diseases with similar pathology but distinct aetiology such as sarcoidosis, pneumonia, and lung cancer (35, 39, 65–71).

Previous work on *M. tuberculosis* and human immunodeficiency virus infection in humans has shown that *CXCR4* is upregulated in blood monocytes and bronchoalveolar lavage cells from human patients with pulmonary TB (72–74). In addition, a loss-of-function mutation in the murine *Thbd* gene that impairs activated protein C production results in uncontrolled lung inflammation in mice infected with *M. tuberculosis*, highlighting the importance of the *THBD* gene in mammalian TB disease (75). Galietti et al. have also shown that *M. tuberculosis–* and *M. bovis*–infected, but not *M. avium*–infected, human monocytes showed increased expression of the CDKN1A protein encoded by *CDKN1A* (76). A range of studies have shown that levels of the protein product of the *PLAUR* gene are elevated in serum from human patients infected with *M. tuberculosis* (77–81). Increased expression of the *OSM* gene and induction of matrix metalloproteinases, which contribute to tissue damage characteristic of TB, have been demonstrated in *M. tuberculosis*–infected human monocytes (82). High expression of *OSM* was also observed in the blood transcriptome of patients presenting with high mycobacterial load sputum (83). In addition, the *OSM* gene is located within a candidate QTL region for TB susceptibility identified using admixture mapping in humans (84).

Ten of the 19 genes that showed consistently increased expression across all post-infection time points were observed to overlap with results from RNA-seq of an *in vitro* infection time-course experiment using bovine AMs stimulated with *M. bovis* (48). *FOSB* and *NR4A1* were upregulated in AMs at 2 hpi; *CXCL8, NR4A1, PLAUR*, and *RGS16* were upregulated at 6 hpi; *EVI2A, CXCL8, FOSB, HBEGF, OSM, PLAUR, RGS16*, and *THBD* were upregulated at 24 hpi; the eight genes observed at 24 hpi plus the *CDKN1A* gene were also upregulated at 48 hpi.

One of the most notable putative transcriptional biomarkers represented in the panel of 19 genes and in the independent PBL studies is *CXCL8* (previously known as *IL8*). CXCL8 is a chemokine encoded by the *CXCL8* gene, which is a strong neutrophil chemoattractant and also chemotactic for monocytes and T cells (85, 86); it has been observed to exhibit increased expression for different mycobacterial infections in a range of mammalian systems (87–95). CXCL8 enhances killing of mycobacteria by neutrophils and macrophages (96, 97), and these immune cells also secrete CXCL8 when stimulated by *M. tuberculosis* (98). In this regard, Godaly and Young showed that *M. bovis* bacillus Calmette–Guérin (BCG) induces CXCL8 secretion by human neutrophils via MyD88-dependent signalling through TLR2 and TLR4 (99). Also, stimulation of human lung fibroblasts *in vitro* using conditioned medium from *M. tuberculosis–*infected monocytes caused prolonged expression of *CXCL8* mRNA and >10-fold increase in CXCL8 secretion (88).

With regard to the *CXCL8* mRNA transcript as a biomarker of infection, Alessandri et al. were able to detect significantly elevated levels of the CXCL8 cytokine in plasma from patients with pulmonary TB (87). More recently, based on reversion of IGRA test results in a Chinese cohort, it has been proposed that decreased serum levels of CXCL8 are associated with clearance of *M. tuberculosis* infection (100). In addition, using microarray and reverse transcriptase–qualitative polymerase chain reaction technologies, Widdison et al. have shown that *M. tuberculosis–* and *M. bovis*–infected bovine AMs express high levels of *CXCL8* transcripts compared to non-infected control cells (90). In support of this, using RNA-seq, we have shown that *CXCL8* increases in expression in bovine AMs infected with either *M. tuberculosis* or *M. bovis* across a 48-h time course (101). *CXCL8* has also been shown to be significantly increased in expression after *in vitro* PPD-b stimulation of PBMCs from cattle infected with *M. bovis* (102) and bovine monocyte-derived macrophages (103). *CXCL8* also exhibited increased expression in PBL (28) but decreased expression in non-stimulated PBMCs from *M. bovis*–infected cattle (104). Also, Almeida de Souza et al. have shown that antimycobacterial treatment reduces high plasma levels of CXCL8 and other CXC chemokines detected in plasma from human patients with active TB (91), and Huang et al. have also demonstrated that AMs and PBMCs from TB patients express CXCL8 at significantly higher levels than healthy controls (94). Interestingly, the potential specificity of increased *CXCL8* gene expression as a biomarker for *M. bovis* infection in cattle is illustrated by recent results obtained by Alonso-Hearn et al. (105). Using similar RNA-seq methodology, they observed significantly decreased expression of *CXCL8* in peripheral blood from cattle infected with *M. avium* subsp. *paratuberculosis*, the causative agent of Johne disease. Finally, it is important to note that several primary studies and meta-analyses have provided evidence that a single-nucleotide polymorphism (rs4073) at the human *CXCL8* gene locus is associated with resistance/susceptibility to *M. tuberculosis* infection (106–109).

### Functional Biology of Peripheral Blood Gene Expression Across the Infection Time Course

Of the 12,406 genes (50.40% of total *B. taurus* reference genes) that were suitable for differential expression analysis, 10,703 genes (86.27%) were mapped to molecules in the IPA Knowledge Base. IPA was used to identify overrepresented canonical pathways and construct biological interaction networks for sets of DE genes at each post-infection time point (+1, +2, +6, +10, and +12 weeks) compared to the pre-infection control time point (−1 week). Only DE genes that were significant after a multiple testing correction was applied (Benjamini–Hochberg method, FDR threshold ≤ 0.05) were used. The gene expression data for the panel of 19 genes (Table 1) were also analysed using IPA and to identify enriched canonical pathways and biological interaction networks. However, these analyses did not reveal any notable functionally relevant pathways or networks (results not shown).

#### +1 Week Post-infection Time Point

Forty-eight of the 57 DE genes detected between sample groups at +1 week post-infection and −1 week pre-infection were mapped to the IPA Knowledge Base (84.21%); however, no statistically significant canonical pathways were detected for this gene expression contrast. Four biological interaction networks were generated from this 57-DE-gene set using the IPA Knowledge Base. Supplementary Figure 6 shows the highest-ranked network, which is associated with embryonic development, organismal development, and reproductive system development and function, and has the ubiquitin C protein encoded by the *UBC* gene as a central hub.

#### +2 Weeks Post-infection Time Point

Eighty-one of the 93 DE genes detected between sample groups at +2 weeks post-infection and −1 week pre-infection were mapped to the IPA Knowledge Base (87.10%). Supplementary Table 8 (Supplementary Material 3) details the overrepresented IPA canonical pathways for this DE gene set. The top-ranked canonical pathway at +2 weeks post-infection was the *Glucocorticoid Receptor Signalling* pathway with eight genes displaying increased expression (*CDKN1A, CXCL8, DUSP1, FOS, IL10, PLAUR, PTGS2*, and *SGK1*) out of a total of 275 members of this pathway (*P* = 1.05 × 10^−5^). The main effects of glucocorticoid steroid hormones signalling through the cytosolic nuclear receptor subfamily 3, group C, member 1 (glucocorticoid receptor) (NR3C1) protein on the immune system are to upregulate expression of anti-inflammatory genes and downregulate expression of proinflammatory genes (110, 111). Therefore, glucocorticoid receptor signalling activity evident in the peripheral blood transcriptome during the early stages of *M. bovis* infection may reflect perturbation of homeostasis (112) and possible modulation of host cellular mechanisms at the site of infection in the lungs.

**Figure 4:**
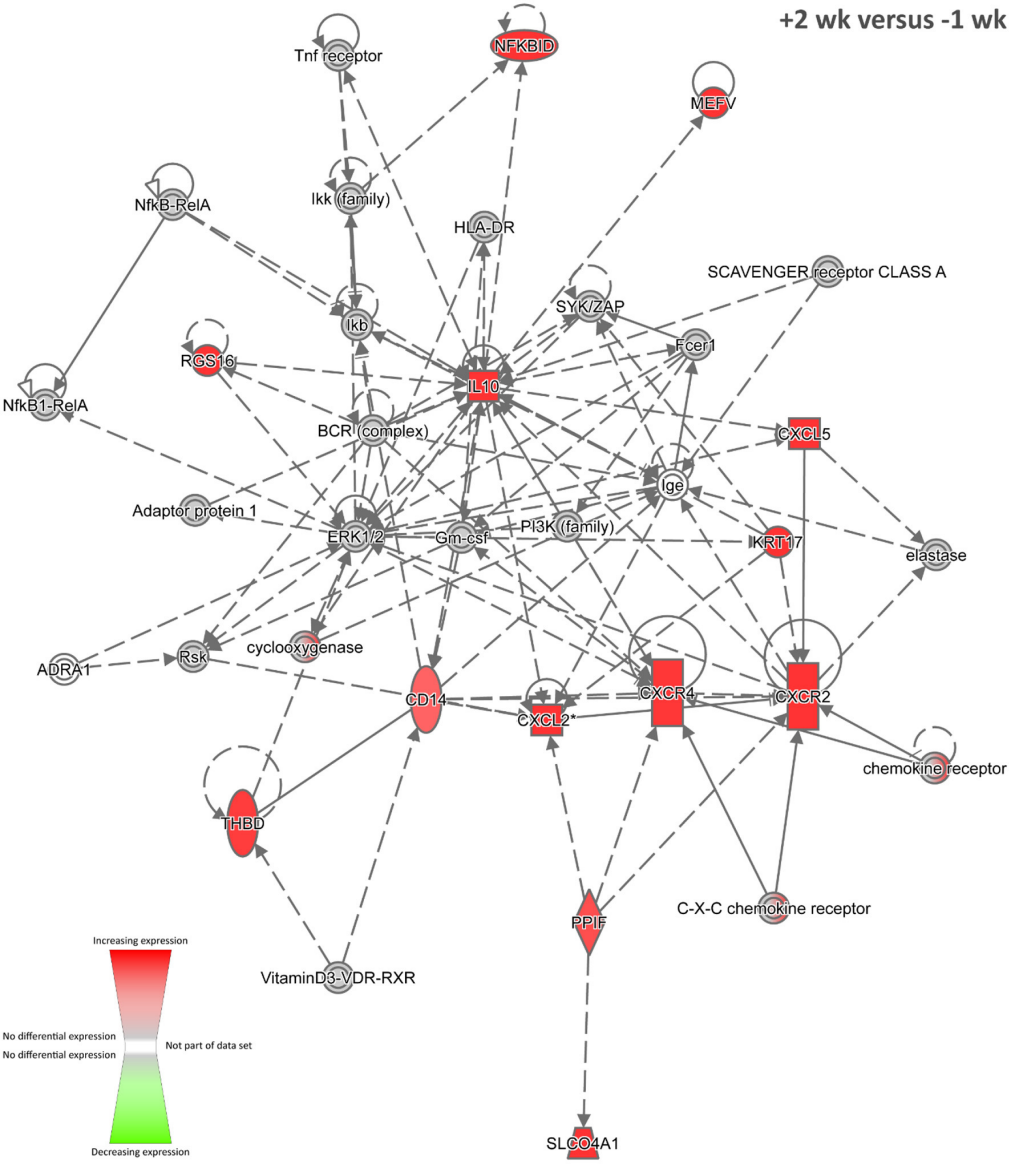
The top-ranked biological interaction network generated using IPA for +2 weeks post-infection. Differential gene expression is represented with a red–green colour scale. This network consisted of 13 focus molecules (IPA Network Score = 24), and the top IPA Disease and Function categories represented were *Cellular Movement, Haematological System Development and Function*, and *Immune Cell Trafficking*. A detailed legend for IPA biological interaction networks including a key for node shapes and edge classifications is available at the following link: https://qiagen.secure.force.com/KnowledgeBase/articles/Basic_Technical_Q_A/Legend.

Nine biological interaction networks were generated from this 81-DE-gene set using the IPA Knowledge Base. Figure 4 shows the highest-ranked network, which was centred on increased expression of the *IL10, CXCL2, CXCR4*, and *CXCR2* genes with *Cellular Movement, Haematological System Development and Function*, and *Immune Cell Trafficking* as the top IPA disease and function categories. In this regard, IL-10, an inhibitory and anti-inflammatory pleiotropic cytokine with a major role in suppression of macrophage and dendritic cell functions, has been hypothesised as a target for modulation and manipulation by mycobacterial pathogens (113, 114). Also, IL-10 is linked to chronic mycobacterial infection in the mouse model (115–117). It has been shown that mycobacterial RNA induces IL-10 production in infected cells through TLR3-mediated activation of the PI3K/AKT signalling pathway (118) and that *M. tuberculosis* infection of THP-1 cells induces *IL10* expression through perturbation of the histone deacetylases HDAC6 and HDAC11 (119). In addition, it has been observed that increased levels of IL-10 cytokine in TB patients lead to impaired T-cell function, thereby contributing to an inefficient host immune response (120).

#### +6 Weeks Post-infection Time Point

Six hundred twenty-six of the 687 DE genes detected between sample groups at +6 weeks post-infection and −1 week pre-infection were mapped to the IPA Knowledge Base (91.12%). Supplementary Table 9 (Supplementary Material 3) details the overrepresented IPA canonical pathways for this DE gene set. Twenty-five biological interaction networks were generated from this 626-DE-gene set using the IPA Knowledge Base, and Supplementary Figure 7 shows the highest-ranked network, which contains mostly down-regulated focus molecules associated with DNA replication, recombination and repair, and control of gene expression and the cell cycle.

#### +10 Weeks Post-infection Time Point

For the +10 weeks post-infection vs. −1 week pre-infection contrast, 2,247 of the 2,583 DE genes were mapped to the IPA Knowledge Base (86.99%). Supplementary Table 10 (Supplementary Material 3) details the overrepresented IPA canonical pathways for this DE gene set. The top-ranked canonical pathway was the *Protein Ubiquitination Pathway*, with 40 of 64 entities present in the pathway containing 255 members (*P* = 1.36 × 10^−11^) exhibiting decreased expression relative to the −1 week pre-infection group. In this regard, it is noteworthy that *M. tuberculosis* has recently been demonstrated to suppress innate immunity by exploiting the host ubiquitination system (121–124).

**Figure 5:**
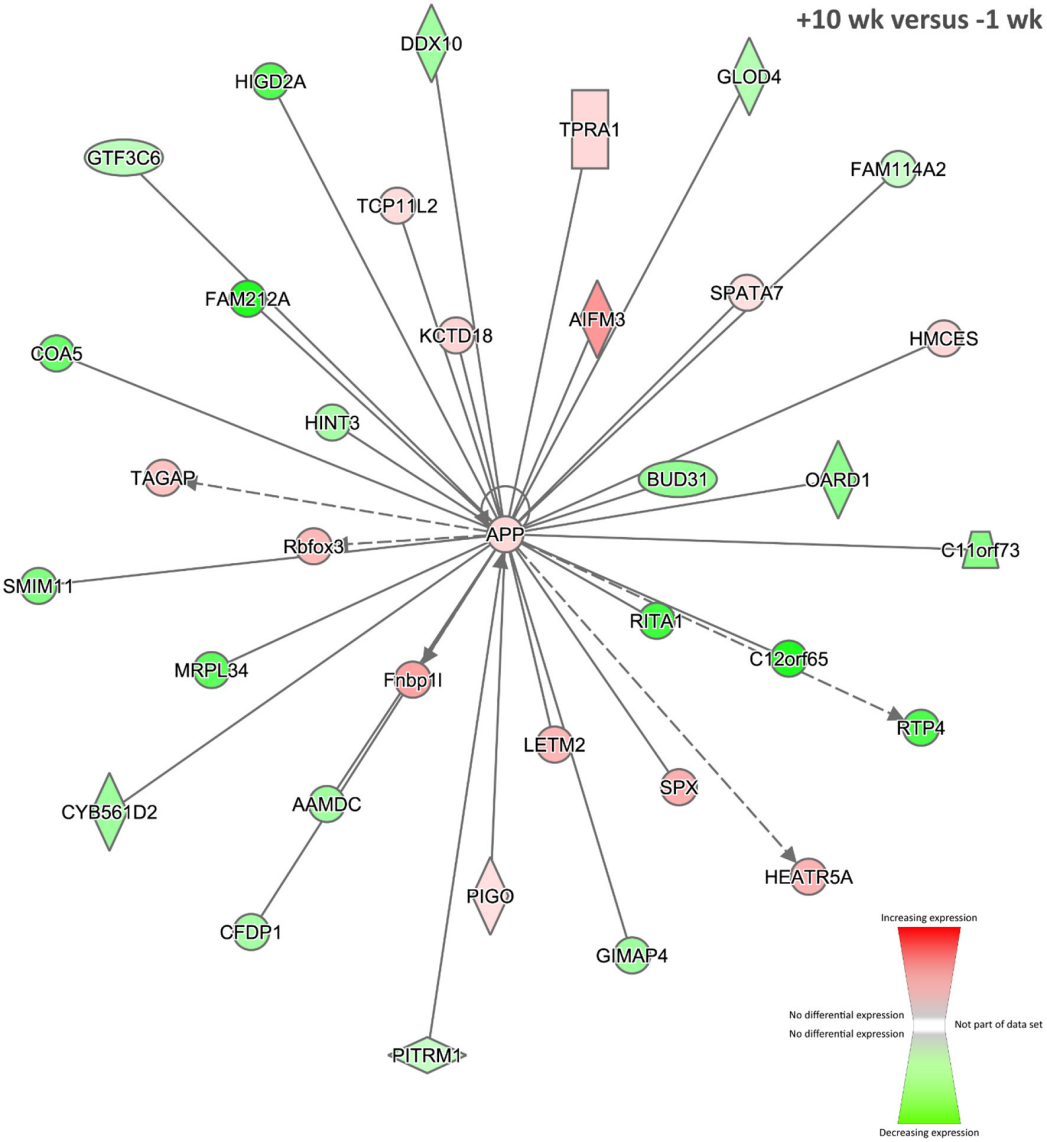
The top-ranked biological interaction network generated using IPA for +10 weeks post-infection. Differential gene expression is represented with a red–green colour scale. This network consisted of 35 focus molecules (IPA Network Score = 39), and the top IPA Disease and Function categories represented were *Antigen Presentation, Carbohydrate Metabolism*, and *Cardiovascular Disease*. A detailed legend for IPA biological interaction networks including a key for node shapes and edge classifications is available at the following link: https://qiagen.secure.force.com/KnowledgeBase/articles/Basic_Technical_Q_A/Legend.

Twenty-five biological interaction networks were generated from this 2,247-DE-gene set using the IPA Knowledge Base. Figure 5 shows the highest-ranked network, which was centred on the amyloid β (A4) precursor protein encoded by the *APP* gene (previously known as *ABPP*) as a central hub, and the top IPA disease and function categories represented were *Antigen Presentation, Carbohydrate Metabolism*, and *Cardiovascular Disease*. Currently, there are few published research works demonstrating differential expression of the *APP* gene during TB in vertebrates (125, 126); however, the presence of this network at +10 weeks post-infection suggests that the represented genes and gene products may have roles in BTB development and host–pathogen interactions. In addition, using RNA-seq, the *APP* gene was also significantly increased in expression in PBL from *M. bovis*–infected cattle compared to non-infected controls (31).

#### +12 Weeks Post-infection Time Point

There was a marked decrease in the number of DE genes at +12 weeks compared to +10 weeks post-infection, which may reflect control of the infection by the immune system at this stage of the time course. Two hundred ninety-three of the 338 DE genes detected between sample groups at +12 weeks post-infection and −1 week pre-infection were mapped to the IPA Knowledge Base (86.69%). Supplementary Table 11 (Supplementary Material 3) details the overrepresented IPA canonical pathways for this DE gene set. The top-ranked canonical pathway was *T Cell Receptor Signalling* with 12 molecules detected from a total of 97 pathway members (*P* = 8.96 × 10^−9^). These 12 genes (*CAMK4, CD247, CD3D, CD3E, CD3G, CD8A, CD8B, FOS, ITK, LCK, PRKCQ*, and *ZAP70*) all displayed increased expression relative to −1 week pre-infection, pointing towards the presence of mycobacterial antigen presentation and T-cell activation via T-cell receptor (TCR) signal transduction (12, 127, 128).

**Figure 6:**
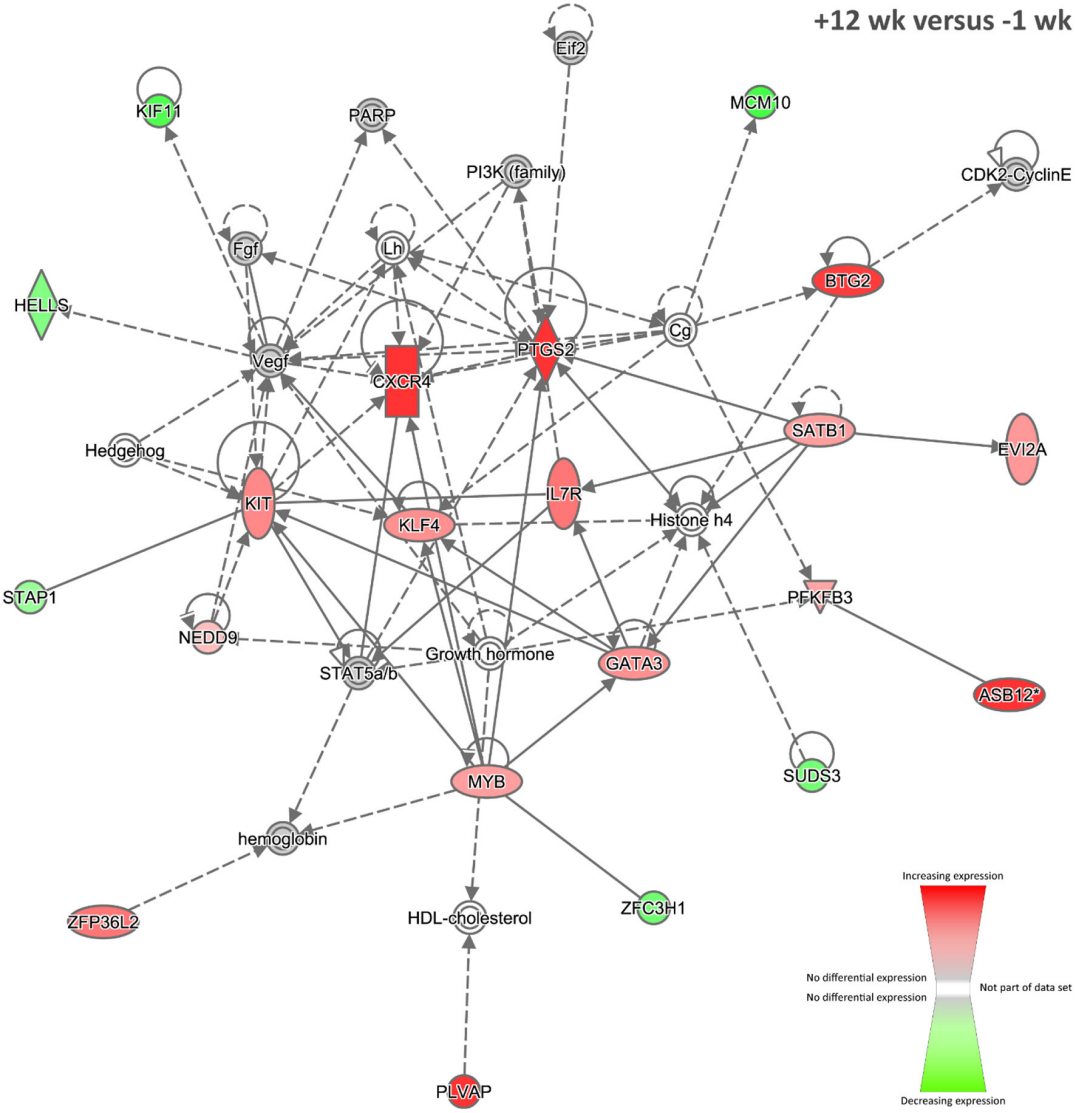
The top-ranked biological interaction network generated using IPA for +12 weeks post-infection. Differential gene expression is represented with a red–green colour scale. This network consisted of 21 focus molecules (IPA Network Score = 33), and the top IPA Disease and Function categories represented were *Cellular Development, Haematological System Development and Function*, and *Cell-mediated Immune Response*. A detailed legend for IPA biological interaction networks including a key for node shapes and edge classifications is available at the following link: https://qiagen.secure.force.com/KnowledgeBase/articles/Basic_Technical_Q_A/Legend.

Nineteen biological interaction networks were generated from this 293-DE-gene set using the IPA Knowledge Base, and Figure 6 shows the highest-ranked network, which was centred on increased expression of the CXCR4, PTGS2, and KLF4 proteins, and the top IPA disease and function categories represented were *Cellular Development, Haematological System Development and Function*, and *Cell-mediated Immune Response*. As described above, the *CXCR4* gene is known to be upregulated in blood monocytes and bronchoalveolar lavage cells from human patients with pulmonary TB (74). In addition, the *PTGS2* gene (previously known as *COX-2*) encodes prostaglandin-endoperoxide synthase 2, a key enzyme in prostaglandin biosynthesis, which is known to be triggered in macrophages—via a TLR2-dependent mechanism—by ESAT-6 proteins secreted by virulent *M. tuberculosis* and *M. bovis* (129). In this regard, it has been hypothesised that induction of PTGS2 may facilitate intracellular mycobacterial survival through inhibition of p53-dependent apoptosis (130). Conversely, it has also been shown that PTGS2 enhances bactericidal activity in *M. tuberculosis–*infected macrophages through promotion of autophagy (131).

The *KLF4* gene encodes a zinc finger–containing transcription factor that regulates macrophage polarisation, displaying increased expression in M2 macrophages and strongly decreased expression in M1 macrophages (132). Integrative network analyses of transcriptome, protein–protein interaction, and transcription factor–binding site data have shown that KLF4 is an important regulator of lung cell gene expression during the early events of *M. tuberculosis* infection in mice (133). It has also been shown that nitric oxide (NO) and KLF4 epigenetically modify class II transactivator protein causing repression of major histocompatibility complex (MHC) class II expression during *M. bovis* BCG infection of murine macrophages (134). Furthermore, downregulation of microRNA-26a during *M. tuberculosis* infection of murine macrophages upregulates KLF4, in turn promoting increased arginase and decreased activity of inducible NO synthase, as well as preventing trafficking of *M. tuberculosis* to lysosomes (135). Taken together, these results support the hypothesis that increased expression of *KLF4* facilitates mycobacterial evasion of host immune surveillance.

### Time-Series Analysis

The STEM tool was designed specifically for analyses of short time-series data sets (3–8 time points) (59, 60) similar to the RNA-gene expression data set obtained from the *M. bovis* animal infection time-course experiment described here. Time-series analysis can be a powerful technique for uncovering networks of coregulated genes in longitudinal time-course experiments (136–138), particularly for gene expression data associated with host immunobiological responses to infection (139–142).

For the present study, STEM time-series analyses of differential gene expression across the *M. bovis* infection time course demonstrated that large groups of genes exhibited comparable patterns of gene expression across the five post-infection time points (+1, +2, +6, +10, and +12 weeks post-infection) relative to the −1 week pre-infection time point. Two different STEM analyses were performed based on (a) expression data for all detectable expressed genes across the infection time course, which corresponded to 4,103 genes (*STEM analysis 1*) and (b) the union set of DE genes from all post-infection time points vs. −1 week pre-infection, which corresponded to 2,935 genes (*STEM analysis 2*). Supplementary Figure 8 shows the top 50 time-series model profiles obtained for *STEM analysis 1* and *STEM analysis 2*.

**Figure 7:**
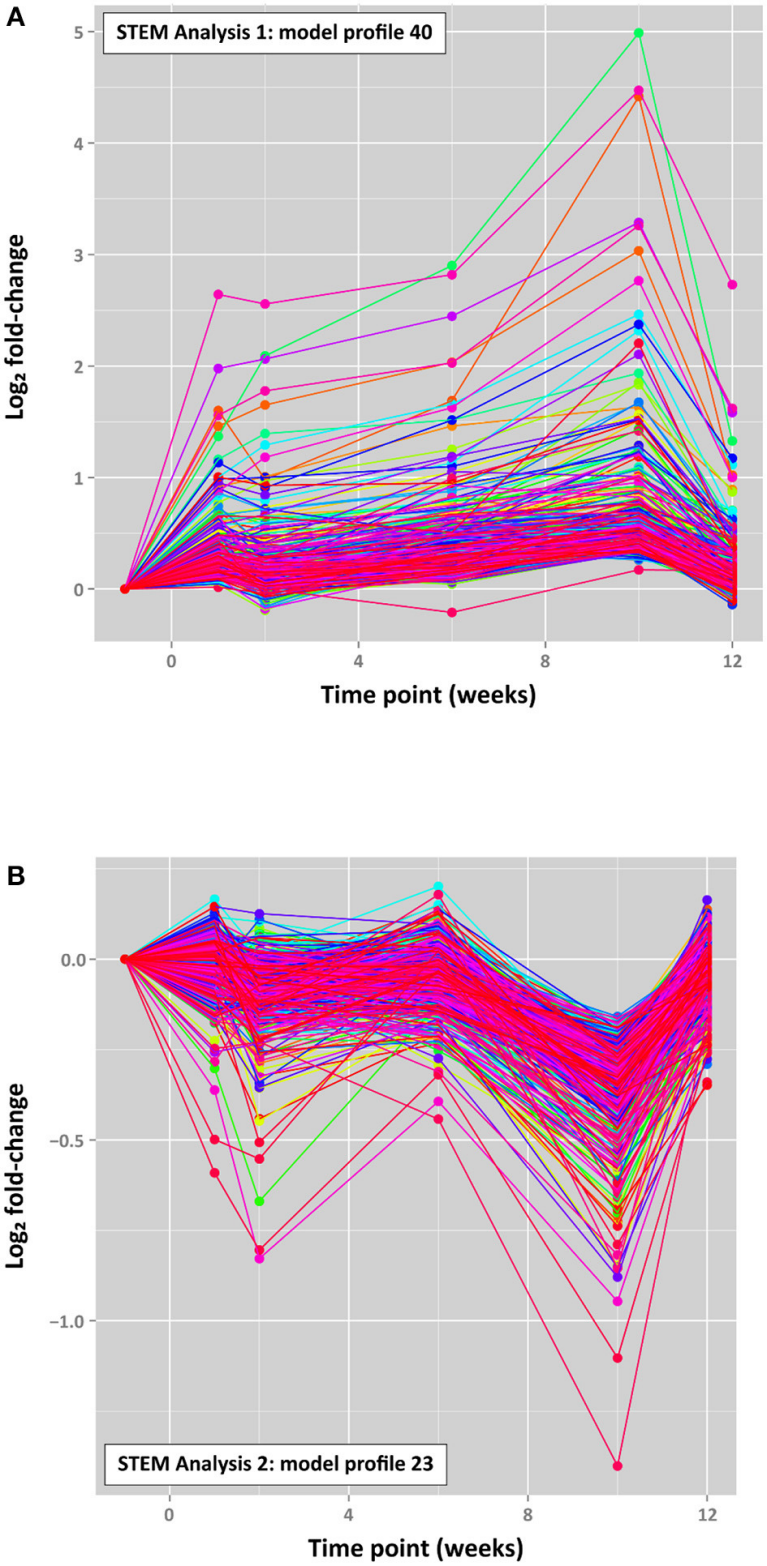
Top-ranked STEM time series profiles. **(A)** The first-ranked model profile (profile 40) for *STEM analysis 1* (4,103 filtered genes) (303 genes assigned; 80.7 genes expected; FDR-adjusted *P* = 3.2 × 10^−99^) and **(B)** the first-ranked model profile (profile 23) for *STEM analysis 2* (2,935 filtered genes) (516 genes assigned; 108.9 genes expected; FDR-adjusted *P* = 1.6 × 10^−187^). The coloured lines in each model profile represent individual genes.

The top-ranked time-series profiles (by *P*-value) obtained using the two different STEM analyses were very similar. For example, as shown in Supplementary Table 12 (Supplementary Material 4), the first-ranked STEM model profile for *STEM analysis 1* (profile 40; Figure 7) was enriched for the *signal transduction, single organism signalling, cell communication, regulation of multicellular organismal process*, and *cellular response to stimulus* GO terms. This profile was also similar to the second-ranked model profile for *STEM analysis 2* (profile 40; Supplementary Figure 9), which was enriched for many of the same GO terms (*signal transduction, single organism signalling, cell communication*; see Supplementary Table 13, Supplementary Material 4). The third-ranked model profile for *STEM analysis 1* (profile 23; Supplementary Figure 9) was highly similar to the first-ranked model profile for *STEM analysis 2* (profile 23; Figure 7) with exactly the same overrepresented GO terms (*mitochondrial inner membrane, organelle inner membrane, mitochondrial part, mitochondrial membrane, mitochondrial envelope*; Supplementary Tables 14, 15, Supplementary Material 4).

It is interesting to note that STEM profile 40 in each analysis is characterised by a cluster of ~300 genes associated with cell signalling and cellular response to stimuli, which exhibited increasing expression across the four time points with a peak at +10 weeks, followed by a substantial decrease at +12 weeks (Figure 7). Conversely, STEM profile 23 (516 genes for *STEM analysis 2*) is characterised by genes associated with mitochondrial components, particularly the mitochondrial membrane, which displayed an oscillating pattern of expression with a marked decrease at +1 week 10 post-infection (Figure 7).

These time-dependent patterns of gene expression in peripheral blood may reflect pathogenesis of early BTB disease during the infection time course with concomitant host cellular responses to *M. bovis* infection, disruption of homeostasis, and changing cellular, tissue, and organismal energy requirements (143–145). In addition, it is important to note that although these longitudinal patterns of gene expression may be due to coregulation of genes in the same cluster, they are likely to also reflect fluctuations in peripheral blood cell type populations comparable to those previously observed for comparisons of *M. bovis*–infected and control non-infected cattle (28, 104).

## Conclusions

The results presented here provide good support for the hypothesis that the peripheral blood transcriptome constitutes a source of gene expression biomarkers for BTB caused by *M. bovis* infection in cattle. This is particularly apparent for the panel of 19 genes exhibiting consistently, statistically significantly increased expression across the infection time course, the majority of which (16 genes) were also significantly increased in PBL harvested from an independent cohort of field-infected cattle. However, the sensitivity and specificity of putative transcriptional biosignatures of *M. bovis* infection will need to be verified and validated using larger panels of cattle naturally infected with *M. bovis* and also populations of animals infected with a range of viral and bacterial pathogens.

## Data Availability Statement

The datasets presented in this study can be found in online repositories. The names of the repository/repositories and accession number(s) can be found at: https://www.ebi.ac.uk/ena, PRJEB27764 and PRJEB44470.

## Ethics Statement

The animal study was reviewed and approved by Animal & Plant Health Agency (APHA), Animal Use Ethics Committee (UK Home Office PCD number 70/6905).

## Author Contributions

DMac, SG, EG, KM, AW, BV-R, and HV conceived and designed the project and organised bovine sample collection. KM, NN, DMag, and JB performed RNA extraction and RNA-seq library generation. KM, NN, KR-A, CC, and DMac performed the analyses. KM, CC, and DMac wrote the manuscript. All authors reviewed and approved the final manuscript.

## Conflict of Interest

The authors declare that the research was conducted in the absence of any commercial or financial relationships that could be construed as a potential conflict of interest.

## References

[1] Cole ST Brosch R Parkhill J Garnier T Churcher C Harris D. Deciphering the biology of *Mycobacterium tuberculosis* from the complete genome sequence. Nature. (1998) 393:537–44. 10.1038/31159 9634230

[2] Garnier T Eiglmeier K Camus JC Medina N Mansoor H Pryor M. The complete genome sequence of *Mycobacterium bovis*. Proc Natl Acad Sci USA. (2003) 100:7877–82. 10.1073/pnas.1130426100 12788972 PMC164681

[3] Brites D Loiseau C Menardo F Borrell S Boniotti MB Warren R. A new phylogenetic framework for the animal-adapted *Mycobacterium tuberculosis* complex. Front Microbiol. (2018) 9:2820. 10.3389/fmicb.2018.02820 30538680 PMC6277475

[4] Malone KM Gordon SV. *Mycobacterium tuberculosis* complex members adapted to wild and domestic animals. Adv Exp Med Biol. (2017) 1019:135–54. 10.1007/978-3-319-64371-7_7 29116633

[5] Perry BD Randolph TF McDermott JJ Sones KR Thornton PK. Investing in Animal Health Research to Alleviate Poverty. Nairobi: ILRI (International Livestock Research Institute) (2002).

[6] Perry B Grace D. The impacts of livestock diseases and their control on growth and development processes that are pro-poor. Philos Trans R Soc Lond B Biol Sci. (2009) 364:2643–55. 10.1098/rstb.2009.0097 19687035 PMC2865091

[7] Steele JH. Introduction (Part 2 Regional and Country Status Reports). In: Thoen CO, Steele JH, editors, Mycobacterium bovis Infection in Animals and Humans. Ames, IA: Iowa State University Press (1995). p. 169–72.

[8] Waters WR Palmer MV Buddle BM Vordermeier HM. Bovine tuberculosis vaccine research: historical perspectives and recent advances. Vaccine. (2012) 30:2611–22. 10.1016/j.vaccine.2012.02.018 22342705

[9] Olea-Popelka F Muwonge A Perera A Dean AS Mumford E Erlacher-Vindel E. Zoonotic tuberculosis in human beings caused by *Mycobacterium bovis* – a call for action. Lancet Infect Dis. (2017) 17:e21–e5. 10.1016/S1473-3099(16)30139-6 27697390

[10] Kanipe C Palmer MV. *Mycobacterium bovis* and you: a comprehensive look at the bacteria, its similarities to *Mycobacterium tuberculosis*, and its relationship with human disease. Tuberculosis. (2020) 125:102006. 10.1016/j.tube.2020.102006 33032093

[11] Luciano SA Roess A. Human zoonotic tuberculosis and livestock exposure in low- and middle-income countries: a systematic review identifying challenges in laboratory diagnosis. Zoonoses Public Health. (2020) 67:97–111. 10.1111/zph.12684 31919980 PMC7027859

[12] O'Garra A Redford PS McNab FW Bloom CI Wilkinson RJ Berry MP. The immune response in tuberculosis. Annu Rev Immunol. (2013) 31:475–527. 10.1146/annurev-immunol-032712-095939 23516984

[13] Domingo M Vidal E Marco A. Pathology of bovine tuberculosis. Res Vet Sci. (2014) 97:S20–9. 10.1016/j.rvsc.2014.03.017 24731532

[14] Pai M Behr MA Dowdy D Dheda K Divangahi M Boehme CC. Tuberculosis. Nat Rev Dis Primers. (2016) 2:16076. 10.1038/nrdp.2016.76 27784885

[15] Scriba TJ Coussens AK Fletcher HA. Human immunology of tuberculosis. Microbiol Spectr. (2016) 4. 10.1128/9781555819569.ch11 27726784

[16] de Chastellier C. The many niches and strategies used by pathogenic mycobacteria for survival within host macrophages. Immunobiology. (2009) 214:526–42. 10.1016/j.imbio.2008.12.005 19261352

[17] Cambier CJ Falkow S Ramakrishnan L. Host evasion and exploitation schemes of *Mycobacterium tuberculosis*. Cell. (2014) 159:1497–509. 10.1016/j.cell.2014.11.024 25525872

[18] Schorey JS Schlesinger LS. Innate immune responses to tuberculosis. Microbiol Spectr. (2016) 4. 10.1128/microbiolspec.TBTB2-0010-2016 28087945

[19] Awuh JA Flo TH. Molecular basis of mycobacterial survival in macrophages. Cell Mol Life Sci. (2017) 74:1625–48. 10.1007/s00018-016-2422-8 27866220 PMC11107535

[20] Martinot AJ. Microbial offense vs host defense: who controls the TB granuloma? Vet Pathol. (2018) 55:14–26. 10.1177/0300985817705177 28749750

[21] Pagan AJ Ramakrishnan L. The formation and function of granulomas. Annu Rev Immunol. (2018) 36:639–65. 10.1146/annurev-immunol-032712-100022 29400999

[22] Cassidy JP. TB or not TB? The granuloma is the question. Vet Pathol. (2019) 56:508–9. 10.1177/0300985819844815 31185878

[23] More SJ Good M. Understanding and managing bTB risk: perspectives from Ireland. Vet Microbiol. (2015) 176:209–18. 10.1016/j.vetmic.2015.01.026 25724333

[24] More SJ. Can bovine TB be eradicated from the Republic of Ireland? Could this be achieved by 2030? Ir Vet J. (2019) 72:3. 10.1186/s13620-019-0140-x 31057791 PMC6485114

[25] Gormley E Doyle MB Fitzsimons T McGill K Collins JD. Diagnosis of *Mycobacterium bovis* infection in cattle by use of the gamma-interferon (Bovigam) assay. Vet Microbiol. (2006) 112:171–9. 10.1016/j.vetmic.2005.11.029 16321478

[26] Allen AR Skuce RA Byrne AW. Bovine tuberculosis in Britain and Ireland - a perfect storm? The confluence of potential ecological and epidemiological impediments to controlling a chronic infectious disease. Front Vet Sci. (2018) 5:109. 10.3389/fvets.2018.00109 29951489 PMC6008655

[27] Vordermeier HM Jones GJ Buddle BM Hewinson RG Villarreal-Ramos B. Bovine tuberculosis in cattle: vaccines, DIVA tests, and host biomarker discovery. Annu Rev Anim Biosci. (2016) 4:87–109. 10.1146/annurev-animal-021815-111311 26884103

[28] Killick KE Browne JA Park SD Magee DA Martin I Meade KG. Genome-wide transcriptional profiling of peripheral blood leukocytes from cattle infected with *Mycobacterium bovis* reveals suppression of host immune genes. BMC Genomics. (2011) 12:611. 10.1186/1471-2164-12-611 22182502 PMC3292584

[29] Aranday-Cortes E Hogarth PJ Kaveh DA Whelan AO Villarreal-Ramos B Lalvani A. Transcriptional profiling of disease-induced host responses in bovine tuberculosis and the identification of potential diagnostic biomarkers. PLoS ONE. (2012) 7:e30626. 10.1371/journal.pone.0030626 22359547 PMC3281027

[30] Blanco FC Soria M Bianco MV Bigi F. Transcriptional response of peripheral blood mononuclear cells from cattle infected with *Mycobacterium bovis*. PLoS ONE. (2012) 7:e41066. 10.1371/journal.pone.0041066 22815916 PMC3397951

[31] McLoughlin KE Nalpas NC Rue-Albrecht K Browne JA Magee DA Killick KE. RNA-seq transcriptional profiling of peripheral blood leukocytes from cattle infected with *Mycobacterium bovis*. Front Immunol. (2014) 5:396. 10.3389/fimmu.2014.00396 25206354 PMC4143615

[32] Klepp LI Eirin ME Garbaccio S Soria M Bigi F Blanco FC. Identification of bovine tuberculosis biomarkers to detect tuberculin skin test and IFNγ release assay false negative cattle. Res Vet Sci. (2019) 122:7–14. 10.1016/j.rvsc.2018.10.016 30447501

[33] Wiarda JE Boggiatto PM Bayles DO Waters WR Thacker TC Palmer MV. Severity of bovine tuberculosis is associated with innate immune-biased transcriptional signatures of whole blood in early weeks after experimental *Mycobacterium bovis* infection. PLoS ONE. (2020) 15:e0239938. 10.1371/journal.pone.0239938 33166313 PMC7652326

[34] Rhodes SG Buddle BM Hewinson RG Vordermeier HM. Bovine tuberculosis: immune responses in the peripheral blood and at the site of active disease. Immunology. (2000) 99:195–202. 10.1046/j.1365-2567.2000.00944.x 10692036 PMC2327153

[35] Blankley S Berry MP Graham CM Bloom CI Lipman M O'Garra A. The application of transcriptional blood signatures to enhance our understanding of the host response to infection: the example of tuberculosis. Philos Trans R Soc Lond B Biol Sci. (2014) 369:20130427. 10.1098/rstb.2013.0427 24821914 PMC4024221

[36] Chaussabel D. Assessment of immune status using blood transcriptomics and potential implications for global health. Semin Immunol. (2015) 27:58–66. 10.1016/j.smim.2015.03.002 25823891

[37] Correia CN Nalpas NC McLoughlin KE Browne JA Gordon SV MacHugh DE. Circulating microRNAs as potential biomarkers of infectious disease. Front Immunol. (2017) 8:118. 10.3389/fimmu.2017.00118 28261201 PMC5311051

[38] Faherty O'Donnell SL Correia CN MacHugh DE. CHAPTER 4: MicroRNAs and mycobacterial infections in humans and domestic animals. In: Peplow PV, Martinez B, Calin GA, Esquela-Kerscher A, editors. MicroRNAs in Diseases and Disorders: Emerging Therapeutic Targets. Cambridge, MK: The Royal Society of Chemistry (2019). p. 105–32. 10.1039/9781788016421-00105

[39] Maertzdorf J McEwen G Weiner III J Tian S Lader E Schriek U. Concise gene signature for point-of-care classification of tuberculosis. EMBO Mol Med. (2016) 8:86–95. 10.15252/emmm.201505790 26682570 PMC4734838

[40] Dean G Whelan A Clifford D Salguero FJ Xing Z Gilbert S. Comparison of the immunogenicity and protection against bovine tuberculosis following immunization by BCG-priming and boosting with adenovirus or protein based vaccines. Vaccine. (2014) 32:1304–10. 10.1016/j.vaccine.2013.11.045 24269321

[41] Dean GS Clifford D Whelan AO Tchilian EZ Beverley PC Salguero FJ. Protection induced by simultaneous subcutaneous and endobronchial vaccination with BCG/BCG and BCG/adenovirus expressing antigen 85A against *Mycobacterium bovis* in cattle. PLoS ONE. (2015) 10:e0142270. 10.1371/journal.pone.0142270 26544594 PMC4636221

[42] Metcalfe HJ Steinbach S Jones GJ Connelley T Morrison WI Vordermeier M. Protection associated with a TB vaccine is linked to increased frequency of Ag85A-specific CD4(+) T cells but no increase in avidity for Ag85A. Vaccine. (2016) 34:4520–5. 10.1016/j.vaccine.2016.07.055 27498622 PMC5009893

[43] Jones GJ Coad M Khatri B Bezos J Parlane NA Buddle BM. Tuberculin skin testing boosts interferon gamma responses to DIVA reagents in *Mycobacterium bovis*-infected cattle. Clin Vaccine Immunol. (2017) 24:e00551–16. 10.1128/CVI.00551-16 28331078 PMC5424240

[44] Salguero FJ Gibson S Garcia-Jimenez W Gough J Strickland TS Vordermeier HM. Differential cell composition and cytokine expression within lymph node granulomas from BCG-vaccinated and non-vaccinated cattle experimentally infected with *Mycobacterium bovis*. Transbound Emerg Dis. (2017) 64:1734–49. 10.1111/tbed.12561 27615603

[45] Malone KM Farrell D Stuber TP Schubert OT Aebersold R Robbe-Austerman S. Updated reference genome sequence and annotation of *Mycobacterium bovis* AF2122/97. Genome Announc. (2017) 5:e00157–17. 10.1128/genomeA.00157-17 28385856 PMC5383904

[46] Whelan A Court P Xing Z Clifford D Hogarth PJ Vordermeier M. Immunogenicity comparison of the intradermal or endobronchial boosting of BCG vaccinates with Ad5-85A. Vaccine. (2012) 30:6294–300. 10.1016/j.vaccine.2012.07.086 22885013

[47] Johnson M Zaretskaya I Raytselis Y Merezhuk Y McGinnis S Madden TL. NCBI BLAST: a better web interface. Nucleic Acids Res. (2008) 36:W5–9. 10.1093/nar/gkn201 18440982 PMC2447716

[48] Nalpas NC Magee DA Conlon KM Browne JA Healy C McLoughlin KE. RNA sequencing provides exquisite insight into the manipulation of the alveolar macrophage by tubercle bacilli. Sci Rep. (2015) 5:13629. 10.1038/srep13629 26346536 PMC4642568

[49] Andrews S. FastQC - A Quality Control Tool for High Throughput Sequence Data. (2016). Available online at: http://www.bioinformatics.babraham.ac.uk/projects/fastqc/

[50] Zimin AV Delcher AL Florea L Kelley DR Schatz MC Puiu D. A whole-genome assembly of the domestic cow, *Bos taurus*. Genome Biol. (2009) 10:R42. 10.1186/gb-2009-10-4-r42 19393038 PMC2688933

[51] Dobin A Davis CA Schlesinger F Drenkow J Zaleski C Jha S. STAR: ultrafast universal RNA-seq aligner. Bioinformatics. (2013) 29:15–21. 10.1093/bioinformatics/bts635 23104886 PMC3530905

[52] Liao Y Smyth GK Shi W. featureCounts: an efficient general purpose program for assigning sequence reads to genomic features. Bioinformatics. (2014) 30:923–30. 10.1093/bioinformatics/btt656 24227677

[53] Robinson MD McCarthy DJ Smyth GK. edgeR: a Bioconductor package for differential expression analysis of digital gene expression data. Bioinformatics. (2010) 26:139–40. 10.1093/bioinformatics/btp616 19910308 PMC2796818

[54] Durinck S Moreau Y Kasprzyk A Davis S De Moor B Brazma A. BioMart and Bioconductor: a powerful link between biological databases and microarray data analysis. Bioinformatics. (2005) 21:3439–40. 10.1093/bioinformatics/bti525 16082012

[55] Robinson MD Oshlack A. A scaling normalization method for differential expression analysis of RNA-seq data. Genome Biol. (2010) 11:R25. 10.1186/gb-2010-11-3-r25 20196867 PMC2864565

[56] Benjamini Y Hochberg Y. Controlling the false discovery rate - a practical and powerful approach to multiple testing. J R Stat Soc Ser B Methodol. (1995) 57:289–300. 10.1111/j.2517-6161.1995.tb02031.x

[57] Kramer A Green J Pollard J Jr Tugendreich S. Causal analysis approaches in ingenuity pathway analysis. Bioinformatics. (2014) 30:523–30. 10.1093/bioinformatics/btt703 24336805 PMC3928520

[58] Agresti A. A survey of exact inference for contingency tables. Statist Sci. (1992) 7:131–53. 10.1214/ss/1177011454

[59] Ernst J Bar-Joseph Z. STEM: a tool for the analysis of short time series gene expression data. BMC Bioinformatics. (2006) 7:191. 10.1186/1471-2105-7-191 16597342 PMC1456994

[60] Ernst J Nau GJ Bar-Joseph Z. Clustering short time series gene expression data. Bioinformatics. (2005) 1:i159–68. 10.1093/bioinformatics/bti1022 15961453

[61] Chaussabel D Quinn C Shen J Patel P Glaser C Baldwin N. A modular analysis framework for blood genomics studies: application to systemic lupus erythematosus. Immunity. (2008) 29:150–64. 10.1016/j.immuni.2008.05.012 18631455 PMC2727981

[62] Chaussabel D Pascual V Banchereau J. Assessing the human immune system through blood transcriptomics. BMC Biol. (2010) 8:84. 10.1186/1741-7007-8-84 20619006 PMC2895587

[63] Hossain MA Yamato O Yamasaki M Otsuka Y Maede Y. Relation between reticulocyte count and characteristics of erythrocyte 5'-nucleotidase in dogs, cats, cattle and humans. J Vet Med Sci. (2003) 65:193–197. 10.1292/jvms.65.193 12655113

[64] Correia CN McLoughlin KE Nalpas NC Magee DA Browne JA Rue-Albrecht K. RNA sequencing (RNA-seq) reveals extremely low levels of reticulocyte-derived globin gene transcripts in peripheral blood from horses (Equus caballus) and cattle (*Bos taurus*). Front Genet. (2018) 9:278. 10.3389/fgene.2018.00278 30154823 PMC6102425

[65] Berry MP Graham CM McNab FW Xu Z Bloch SA Oni T. An interferon-inducible neutrophil-driven blood transcriptional signature in human tuberculosis. Nature. (2010) 466:973–7. 10.1038/nature09247 20725040 PMC3492754

[66] Maertzdorf J Weiner J. III Mollenkopf HJ Bornot TB Network BT. Common patterns and disease-related signatures in tuberculosis and sarcoidosis. Proc Natl Acad Sci USA. (2012) 109:7853–8. 10.1073/pnas.1121072109 22547807 PMC3356621

[67] Cliff JM Kaufmann SH McShane H van Helden P O'Garra A. The human immune response to tuberculosis and its treatment: a view from the blood. Immunol Rev. (2015) 264:88–102. 10.1111/imr.12269 25703554 PMC4368415

[68] Blankley S Graham CM Turner J Berry MP Bloom CI Xu Z. The transcriptional signature of active tuberculosis reflects symptom status in extra-pulmonary and pulmonary tuberculosis. PLoS ONE. (2016) 11:e0162220. 10.1371/journal.pone.0162220 27706152 PMC5051928

[69] Sweeney TE Braviak L Tato CM Khatri P. Genome-wide expression for diagnosis of pulmonary tuberculosis: a multicohort analysis. Lancet Respir Med. (2016) 4:213–24. 10.1016/S2213-2600(16)00048-5 26907218 PMC4838193

[70] Leong S Zhao Y Joseph NM Hochberg NS Sarkar S Pleskunas J. Existing blood transcriptional classifiers accurately discriminate active tuberculosis from latent infection in individuals from south India. Tuberculosis. (2018) 109:41–51. 10.1016/j.tube.2018.01.002 29559120

[71] Estévez O Anibarro L Garet E Pallares Á Barcia L Calviño L. An RNA-seq based machine learning approach identifies latent tuberculosis patients with an active tuberculosis profile. Front Immunol. (2020) 11:1470. 10.3389/fimmu.2020.01470 32760401 PMC7372107

[72] Hoshino Y Tse DB Rochford G Prabhakar S Hoshino S Chitkara N. *Mycobacterium tuberculosis*-induced CXCR4 and chemokine expression leads to preferential X4 HIV-1 replication in human macrophages. J Immunol. (2004) 172:6251–8. 10.4049/jimmunol.172.10.6251 15128813

[73] Rosas-Taraco AG Arce-Mendoza AY Caballero-Olin G Salinas-Carmona MC. *Mycobacterium tuberculosis* upregulates coreceptors CCR5 and CXCR4 while HIV modulates CD14 favoring concurrent infection. AIDS Res Hum Retroviruses. (2006) 22:45–51. 10.1089/aid.2006.22.45 16438645

[74] Shankar EM Vignesh R Ellegard R Barathan M Chong YK Bador MK. HIV-*Mycobacterium tuberculosis* co-infection: a 'danger-couple model' of disease pathogenesis. Pathog Dis. (2014) 70:110–8. 10.1111/2049-632X.12108 24214523

[75] Weijer S Wieland CW Florquin S van der Poll T. A thrombomodulin mutation that impairs activated protein C generation results in uncontrolled lung inflammation during murine tuberculosis. Blood. (2005) 106:2761–8. 10.1182/blood-2004-12-4623 16014564

[76] Galietti F Bollo E Cappia S Dondo A Pregel P Nicali R. p53 expression in cultured blood human monocytes infected with mycobacterial strains. Panminerva Med. (2001) 43:249–55. 11677419

[77] Juffermans NP Dekkers PE Verbon A Speelman P van Deventer SJ van der Poll T. Concurrent upregulation of urokinase plasminogen activator receptor and CD11b during tuberculosis and experimental endotoxemia. Infect Immun. (2001) 69:5182–5. 10.1128/IAI.69.8.5182-5185.2001 11447203 PMC98617

[78] Eugen-Olsen J Gustafson P Sidenius N Fischer TK Parner J Aaby P. The serum level of soluble urokinase receptor is elevated in tuberculosis patients and predicts mortality during treatment: a community study from Guinea-Bissau. Int J Tuberc Lung Dis. (2002) 6:686–92. 12150480

[79] Ostrowski SR Ravn P Hoyer-Hansen G Ullum H Andersen AB. Elevated levels of soluble urokinase receptor in serum from mycobacteria infected patients: still looking for a marker of treatment efficacy. Scand J Infect Dis. (2006) 38:1028–32. 10.1080/00365540600868305 17148072

[80] Djoba Siawaya JF Bapela NB Ronacher K Veenstra H Kidd M Gie R. Immune parameters as markers of tuberculosis extent of disease and early prediction of anti-tuberculosis chemotherapy response. J Infect. (2008) 56:340–7. 10.1016/j.jinf.2008.02.007 18359089

[81] Araujo Z Macias-Segura N Lopez-Ramos JE De Waard JH Vanegas M Patarroyo MA. Diagnostic accuracy of combinations of serological biomarkers for identifying clinical tuberculosis. J Infect Dev Ctries. (2018) 12:429–41. 10.3855/jidc.9554 31940294

[82] O'Kane CM Elkington PT Friedland JS. Monocyte-dependent oncostatin M and TNF-alpha synergize to stimulate unopposed matrix metalloproteinase-1/3 secretion from human lung fibroblasts in tuberculosis. Eur J Immunol. (2008) 38:1321–30. 10.1002/eji.200737855 18398932

[83] Dupnik KM Bean JM Lee MH Jean Juste MA Skrabanek L Rivera V. Blood transcriptomic markers of *Mycobacterium tuberculosis* load in sputum. Int J Tuberc Lung Dis. (2018) 22:950–8. 10.5588/ijtld.17.0855 29991407 PMC6343854

[84] Daya M van der Merwe L Gignoux CR van Helden PD Moller M Hoal EG. Using multi-way admixture mapping to elucidate TB susceptibility in the South African Coloured population. BMC Genomics. (2014) 15:1021. 10.1186/1471-2164-15-1021 25422094 PMC4256931

[85] Zhang Y Broser M Cohen H Bodkin M Law K Reibman J. Enhanced interleukin-8 release and gene expression in macrophages after exposure to *Mycobacterium tuberculosis* and its components. J Clin Invest. (1995) 95:586–92. 10.1172/JCI117702 7860742 PMC295520

[86] Gerszten RE Garcia-Zepeda EA Lim YC Yoshida M Ding HA Gimbrone. MCP-1 and IL-8 trigger firm adhesion of monocytes to vascular endothelium under flow conditions. Nature. (1999) 398:718–23. 10.1038/19546 10227295

[87] Alessandri AL Souza AL Oliveira SC Macedo GC Teixeira MM Teixeira AL. Concentrations of CXCL8, CXCL9 and sTNFR1 in plasma of patients with pulmonary tuberculosis undergoing treatment. Inflamm Res. (2006) 55:528–33. 10.1007/s00011-006-5136-9 17039284

[88] O'Kane CM Boyle JJ Horncastle DE Elkington PT Friedland JS. Monocyte-dependent fibroblast CXCL8 secretion occurs in tuberculosis and limits survival of mycobacteria within macrophages. J Immunol. (2007) 178:3767–76. 10.4049/jimmunol.178.6.3767 17339475

[89] Sawant KV McMurray DN. Guinea pig neutrophils infected with *Mycobacterium tuberculosis* produce cytokines which activate alveolar macrophages in noncontact cultures. Infect Immun. (2007) 75:1870–7. 10.1128/IAI.00858-06 17283104 PMC1865707

[90] Widdison S Watson M Piercy J Howard C Coffey TJ. Granulocyte chemotactic properties of *M. tuberculosis* versus *M. bovis-*infected bovine alveolar macrophages. Mol Immunol. (2008) 45:740–9. 10.1016/j.molimm.2007.06.357 17698194

[91] Almeida de Souza C Abramo C Alves CC Mazzoccoli L Ferreira AP Teixeira HC. Anti-mycobacterial treatment reduces high plasma levels of CXC-chemokines detected in active tuberculosis by cytometric bead array. Mem Inst Oswaldo Cruz. (2009) 104:1039–41. 10.1590/S0074-02762009000700018 20027475

[92] Lee HM Shin DM Kim KK Lee JS Paik TH Jo EK. Roles of reactive oxygen species in CXCL8 and CCL2 expression in response to the 30-kDa antigen of *Mycobacterium tuberculosis*. J Clin Immunol. (2009) 29:46–56. 10.1007/s10875-008-9222-3 18690522

[93] Andersson M Lutay N Hallgren O Westergren-Thorsson G Svensson M Godaly G. *Mycobacterium bovis* bacilli Calmette-Guerin regulates leukocyte recruitment by modulating alveolar inflammatory responses. Innate Immun. (2012) 18:531–40. 10.1177/1753425911426591 22058091 PMC3548393

[94] Huang KH Wang CH Lee KY Lin SM Lin CH Kuo HP. NF-kappaB repressing factor inhibits chemokine synthesis by peripheral blood mononuclear cells and alveolar macrophages in active pulmonary tuberculosis. PLoS ONE. (2013) 8:e77789. 10.1371/journal.pone.0077789 24223729 PMC3817197

[95] Domingo-Gonzalez R Prince O Cooper A Khader SA. Cytokines and chemokines in *Mycobacterium tuberculosis* infection. Microbiol Spectr. (2016) 4. 10.1128/microbiolspec.TBTB2-0018-2016 27763255 PMC5205539

[96] Nibbering PH Pos O Stevenhagen A Van Furth R. Interleukin-8 enhances nonoxidative intracellular killing of *Mycobacterium fortuitum* by human granulocytes. Infect Immun. (1993) 61:3111–6. 10.1128/IAI.61.8.3111-3116.1993 8335340 PMC280976

[97] Krupa A Fol M Dziadek BR Kepka E Wojciechowska D Brzostek A. Binding of CXCL8/IL-8 to *Mycobacterium tuberculosis* modulates the innate immune response. Mediators Inflamm. (2015) 2015:124762. 10.1155/2015/124762 26300588 PMC4537748

[98] Kasahara K Sato I Ogura K Takeuchi H Kobayashi K Adachi M. Expression of chemokines and induction of rapid cell death in human blood neutrophils by *Mycobacterium tuberculosis*. J Infect Dis. (1998) 178:127–137. 10.1086/515585 9652432

[99] Godaly G Young DB. *Mycobacterium bovis* bacille Calmette Guerin infection of human neutrophils induces CXCL8 secretion by MyD88-dependent TLR2 and TLR4 activation. Cell Microbiol. (2005) 7:591–601. 10.1111/j.1462-5822.2004.00489.x 15760459

[100] Xin H Zhang H Cao X Li X Li M Feng B. Serum level of IL-8 is associated with reversion of QuantiFERON-TB gold in-tube tests. J Infect. (2019) 78:292–8. 10.1016/j.jinf.2018.08.010 30138640

[101] Malone KM Rue-Albrecht K Magee DA Conlon K Schubert OT Nalpas NC. Comparative 'omics analyses differentiate *Mycobacterium tuberculosis* and *Mycobacterium bovis* and reveal distinct macrophage responses to infection with the human and bovine tubercle bacilli. Microb Genom. (2018) 4:e000163. 10.1099/mgen.0.000163 29557774 PMC5885015

[102] Meade KG Gormley E O'Farrelly C Park SD Costello E Keane J. Antigen stimulation of peripheral blood mononuclear cells from *Mycobacterium bovis* infected cattle yields evidence for a novel gene expression program. BMC Genomics. (2008) 9:447. 10.1186/1471-2164-9-447 18823559 PMC2569068

[103] Taraktsoglou M Szalabska U Magee DA Browne JA Sweeney T Gormley E. Transcriptional profiling of immune genes in bovine monocyte-derived macrophages exposed to bacterial antigens. Vet Immunol Immunopathol. (2011) 140:130–9. 10.1016/j.vetimm.2010.12.002 21242003

[104] Meade KG Gormley E Doyle MB Fitzsimons T O'Farrelly C Costello E. Innate gene repression associated with *Mycobacterium bovis* infection in cattle: toward a gene signature of disease. BMC Genomics. (2007) 8:400. 10.1186/1471-2164-8-400 17974019 PMC2213678

[105] Alonso-Hearn M Canive M Blanco-Vazquez C Torremocha R Balseiro A Amado J. RNA-Seq analysis of ileocecal valve and peripheral blood from Holstein cattle infected with Mycobacterium avium subsp. paratuberculosis revealed dysregulation of the CXCL8/IL8 signaling pathway. Sci Rep. (2019) 9:14845. 10.1038/s41598-019-51328-0 31619718 PMC6795908

[106] Ma X Reich RA Wright JA Tooker HR Teeter LD Musser JM. Association between interleukin-8 gene alleles and human susceptibility to tuberculosis disease. J Infect Dis. (2003) 188:349–55. 10.1086/376559 12870115

[107] Lindenau JD Guimarães LS Friedrich DC Hurtado AM Hill KR Salzano FM. Cytokine gene polymorphisms are associated with susceptibility to tuberculosis in an Amerindian population. Int J Tuberc Lung Dis. (2014) 18:952–7. 10.5588/ijtld.14.0060 25199010

[108] Yu Z Wit W Xiong L Cheng Y. Associations of six common functional polymorphisms in interleukins with tuberculosis: evidence from a meta-analysis. Pathog Dis. (2019) 77:ftz053. 10.1093/femspd/ftz053 31560754

[109] Chen J Ma A. Associations of polymorphisms in interleukins with tuberculosis: evidence from a meta-analysis. Immunol Lett. (2020) 217:1–6. 10.1016/j.imlet.2019.10.012 31669382

[110] Rogatsky I Ivashkiv LB. Glucocorticoid modulation of cytokine signaling. Tissue Antigens. (2006) 68:1–12. 10.1111/j.1399-0039.2006.00599.x 16774534

[111] Busillo JM Cidlowski JA. The five Rs of glucocorticoid action during inflammation: ready, reinforce, repress, resolve, and restore. Trends Endocrinol Metab. (2013) 24:109–19. 10.1016/j.tem.2012.11.005 23312823 PMC3667973

[112] D'Attilio L Santucci N Bongiovanni B Bay ML Bottasso O. Tuberculosis, the disrupted immune-endocrine response and the potential thymic repercussion as a contributing factor to disease physiopathology. Front Endocrinol. (2018) 9:214. 10.3389/fendo.2018.00214 29765355 PMC5938357

[113] Redford PS Murray PJ O'Garra A. The role of IL-10 in immune regulation during *M. tuberculosis* infection. Mucosal Immunol. (2011) 4:261–70. 10.1038/mi.2011.7 21451501

[114] Hmama Z Pena-Diaz S Joseph S Av-Gay Y. Immunoevasion and immunosuppression of the macrophage by *Mycobacterium tuberculosis*. Immunol Rev. (2015) 264:220–32. 10.1111/imr.12268 25703562

[115] Turner J Gonzalez-Juarrero M Ellis DL Basaraba RJ Kipnis A Orme IM. *In vivo* IL-10 production reactivates chronic pulmonary tuberculosis in C57BL/6 mice. J Immunol. (2002) 169:6343–51. 10.4049/jimmunol.169.11.6343 12444141

[116] Huynh JP Lin CC Kimmey JM Jarjour NN Schwarzkopf EA Bradstreet TR. Bhlhe40 is an essential repressor of IL-10 during *Mycobacterium tuberculosis* infection. J Exp Med. (2018) 215:1823–38. 10.1084/jem.20171704 29773644 PMC6028511

[117] Ouyang W O'Garra A. IL-10 family cytokines IL-10 and IL-22: from basic science to clinical translation. Immunity. (2019) 50:871–91. 10.1016/j.immuni.2019.03.020 30995504

[118] Bai W Liu H Ji Q Zhou Y Liang L Zheng R. TLR3 regulates mycobacterial RNA-induced IL-10 production through the PI3K/AKT signaling pathway. Cell Signal. (2014) 26:942–50. 10.1016/j.cellsig.2014.01.015 24462705

[119] Wang X Wu Y Jiao J Huang Q. *Mycobacterium tuberculosis* infection induces IL-10 gene expression by disturbing histone deacetylase 6 and histone deacetylase 11 equilibrium in macrophages. Tuberculosis. (2018) 108:118–23. 10.1016/j.tube.2017.11.008 29523311

[120] Harling K Adankwah E Guler A Afum-Adjei Awuah A Adu-Amoah L Mayatepek E. Constitutive STAT3 phosphorylation and IL-6/IL-10 co-expression are associated with impaired T-cell function in tuberculosis patients. Cell Mol Immunol. (2019) 16:275–87. 10.1038/cmi.2018.5 30886421 PMC6460487

[121] Wang J Li BX Ge PP Li J Wang Q Gao GF. *Mycobacterium tuberculosis* suppresses innate immunity by coopting the host ubiquitin system. Nat Immunol. (2015) 16:237–45. 10.1038/ni.3096 25642820

[122] Franco LH Nair VR Scharn CR Xavier RJ Torrealba JR Shiloh MU. The ubiquitin ligase Smurf1 functions in selective autophagy of *Mycobacterium tuberculosis* and anti-tuberculous host defense. Cell Host Microbe. (2017) 21:59–72. 10.1016/j.chom.2016.11.002 28017659 PMC5699477

[123] Chai Q Wang X Qiang L Zhang Y Ge P Lu Z. A *Mycobacterium tuberculosis* surface protein recruits ubiquitin to trigger host xenophagy. Nat Commun. (2019) 10:1973. 10.1038/s41467-019-09955-8 31036822 PMC6488588

[124] Chai Q Wang L Liu CH Ge B. New insights into the evasion of host innate immunity by *Mycobacterium tuberculosis*. Cell Mol Immunol. (2020) 17:901–13. 10.1038/s41423-020-0502-z 32728204 PMC7608469

[125] Wang JP Rought SE Corbeil J Guiney DG. Gene expression profiling detects patterns of human macrophage responses following *Mycobacterium tuberculosis* infection. FEMS Immunol Med Microbiol. (2003) 39:163–72. 10.1016/S0928-8244(03)00223-2 14625100

[126] Keller C Hoffmann R Lang R Brandau S Hermann C Ehlers S. Genetically determined susceptibility to tuberculosis in mice causally involves accelerated and enhanced recruitment of granulocytes. Infect Immun. (2006) 74:4295–309. 10.1128/IAI.00057-06 16790804 PMC1489748

[127] Harding CV Boom WH. Regulation of antigen presentation by *Mycobacterium tuberculosis*: a role for Toll-like receptors. Nat Rev Microbiol. (2010) 8:296–307. 10.1038/nrmicro2321 20234378 PMC3037727

[128] Harriff MJ Purdy GE Lewinsohn DM. Escape from the phagosome: the explanation for MHC-I processing of mycobacterial antigens? Front Immunol. (2012) 3:40. 10.3389/fimmu.2012.00040 22566923 PMC3342008

[129] A SK Bansal K Holla S Verma-Kumar S Sharma P Balaji KN. ESAT-6 induced COX-2 expression involves coordinated interplay between PI3K and MAPK signaling. Mol Immunol. (2012) 49:655–63. 10.1016/j.molimm.2011.11.011 22154837

[130] Dutta NK Mehra S Martinez AN Alvarez X Renner NA Morici LA. The stress-response factor SigH modulates the interaction between *Mycobacterium tuberculosis* and host phagocytes. PLoS ONE. (2012) 7:e28958. 10.1371/journal.pone.0028958 22235255 PMC3250399

[131] Xiong W Wen Q Du X Wang J He W Wang R. Novel function of cyclooxygenase-2: suppressing mycobacteria by promoting autophagy via the protein kinase B/mammalian target of rapamycin pathway. J Infect Dis. (2018) 217:1267–79. 10.1093/infdis/jiy033 29373690

[132] Liao X Sharma N Kapadia F Zhou G Lu Y Hong H. Kruppel-like factor 4 regulates macrophage polarization. J Clin Invest. (2011) 121:2736–49. 10.1172/JCI45444 21670502 PMC3223832

[133] Yaqubi M Mohammadnia A Fallahi H. Transcription factor regulatory network for early lung immune response to tuberculosis in mice. Mol Med Rep. (2015) 12:2865–71. 10.3892/mmr.2015.3721 25955085

[134] Ghorpade DS Holla S Sinha AY Alagesan SK Balaji KN. Nitric oxide and KLF4 protein epigenetically modify class II transactivator to repress major histocompatibility complex II expression during *Mycobacterium bovis* bacillus Calmette-Guerin infection. J Biol Chem. (2013) 288:20592–606. 10.1074/jbc.M113.472183 23733190 PMC3711323

[135] Sahu SK Kumar M Chakraborty S Banerjee SK Kumar R Gupta P. MicroRNA 26a (miR-26a)/KLF4 and CREB-C/EBPβ regulate innate immune signaling, the polarization of macrophages and the trafficking of *Mycobacterium tuberculosis* to lysosomes during infection. PLoS Pathog. (2017) 13:e1006410. 10.1371/journal.ppat.1006410 28558034 PMC5466338

[136] Bar-Joseph Z. Analyzing time series gene expression data. Bioinformatics. (2004) 20:2493–503. 10.1093/bioinformatics/bth283 15130923

[137] Bar-Joseph Z Gitter A Simon I. Studying and modelling dynamic biological processes using time-series gene expression data. Nat Rev Genet. (2012) 13:552–64. 10.1038/nrg3244 22805708

[138] Oh S Song S Dasgupta N Grabowski G. The analytical landscape of static and temporal dynamics in transcriptome data. Front Genet. (2014) 5:35. 10.3389/fgene.2014.00035 24600473 PMC3929947

[139] Blanc M Hsieh WY Robertson KA Watterson S Shui G Lacaze P. Host defense against viral infection involves interferon mediated down-regulation of sterol biosynthesis. PLoS Biol. (2011) 9:e1000598. 10.1371/journal.pbio.1000598 21408089 PMC3050939

[140] Jorgensen HB Buitenhuis B Rontved CM Jiang L Ingvartsen KL Sorensen P. Transcriptional profiling of the bovine hepatic response to experimentally induced *E. coli* mastitis. Physiol Genomics. (2012) 44:595–606. 10.1152/physiolgenomics.00084.2011 22496490

[141] Dimitrakopoulou K Vrahatis AG Wilk E Tsakalidis AK Bezerianos A. OLYMPUS: an automated hybrid clustering method in time series gene expression. Case study: host response after Influenza A (H1N1) infection. Comput Methods Programs Biomed. (2013) 111:650–61. 10.1016/j.cmpb.2013.05.025 23796450

[142] Maslove DM Wong HR. Gene expression profiling in sepsis: timing, tissue, translational considerations. Trends Mol Med. (2014) 20:204–13. 10.1016/j.molmed.2014.01.006 24548661 PMC3976710

[143] Neill SD Bryson DG Pollock JM. Pathogenesis of tuberculosis in cattle. Tuberculosis. (2001) 81:79–86. 10.1054/tube.2000.0279 11463227

[144] Cassidy JP. The pathogenesis and pathology of bovine tuberculosis with insights from studies of tuberculosis in humans and laboratory animal models. Vet Microbiol. (2006) 112:151–161. 10.1016/j.vetmic.2005.11.031 16310979

[145] Waters WR Maggioli MF McGill JL Lyashchenko KP Palmer MV. Relevance of bovine tuberculosis research to the understanding of human disease: historical perspectives, approaches, immunologic mechanisms. Vet Immunol Immunopathol. (2014) 159:113–32. 10.1016/j.vetimm.2014.02.009 24636301

