# Supplementary material for: RNA-Seq Transcriptome Analysis of Peripheral Blood From Cattle Infected With Mycobacterium bovis Across an Experimental Time Course
Source: Front Vet Sci. 2021 May 28;8:662002. doi: 10.3389/fvets.2021.662002 (PMC8193354; doi:10.3389/fvets.2021.662002)
Supplement: Supplementary file 1 [file Data_Sheet_1.DOCX]

Supplementary Material

RNA‐seq transcriptome analysis of peripheral blood from cattle infected with *Mycobacterium bovis* across an experimental time course

**Kirsten E. McLoughlin^1^, Carolina N. Correia^1^, John A. Browne^1^, David A. Magee^1^, Nicolas C. Nalpas^1, †^, Kevin Rue-Albrecht^1, §^, Adam O. Whelan^2,^** ^‡^**, Bernardo Villarreal-Ramos^2, ¶^, H. Martin Vordermeier^2, ¶^, Eamonn Gormley^3^, Stephen V. Gordon^3, 4^, David E. MacHugh^1, 4^***

^1^ Animal Genomics Laboratory, UCD School of Agriculture and Food Science, UCD College of Health and Agricultural Sciences, University College Dublin, Belfield, Dublin, D04 V1W8, Ireland.

^2^ TB Immunology and Vaccinology Team, Department of Bacteriology, Animal and Plant Health Agency, Weybridge, Surrey, KT15 3NB, United Kingdom.

^3^ UCD School of Veterinary Medicine, UCD College of Health and Agricultural Sciences, University College Dublin, Belfield, Dublin, D04 V1W8, Ireland.

^4^ UCD Conway Institute of Biomolecular and Biomedical Research, University College Dublin, Belfield, Dublin, D04 V1W8, Ireland.

^†^ Current address: Quantitative Proteomics and Proteome Centre Tübingen, Interfaculty Institute for Cell Biology, University of Tübingen, Tübingen, 72076, Germany.

^§^ Current address: MRC WIMM Centre for Computational Biology, University of Oxford, Oxford, OX3 9DS, UK.

^‡^ Current address: Biomedical Sciences, Defence Science and Technology Laboratory, Salisbury, Wiltshire, SP4 0JQ, United Kingdom.

**^¶^** BVR and HMV have positions as Ser Cymru II Professors of Immunology at the Institute of Biological, Environmental & Rural Sciences, Aberystwyth University, Penglais, Aberystwyth, Ceredigion, SY23 3FD, United Kingdom.

Correspondence

D. E. MacHugh, Animal Genomics Laboratory, UCD School of Agriculture and Food Science, University College Dublin, Belfield, Dublin, D04 V1W8, Ireland.

Email: [david.machugh@ucd.ie](mailto:david.machugh@ucd.ie)

Phone: +353 1 716 6256

**Note:** British English language style preferred for publication.

Supplementary Tables

**Table S1:** Animal infection time course sample information with total RNA sample quality and quantity data and RNA-seq library pool and barcode index information (contained in Excel file – **Supplementary Information File 1**).

**Table S2:** Filtering and mapping statistics for 52 RNA-seq libraries from the animal infection time course experiment (-1 wk pre-infection and +1 wk, +2 wk, +6 wk, +10 wk and +12 wk) (contained in Excel file – **Supplementary Information File 1**).

**Table S3:** DE genes showing **(a)** increased and **(b)** decreased expression at +1 wk post-infection relative to the -1 wk pre-infection control group (ranked according to *P*-value [smallest to largest]) (contained in Excel file – **Supplementary Information File 2**).

**Table S4:** DE genes showing **(a)** increased and **(b)** decreased expression at +2 wk post-infection relative to the -1 wk pre-infection control group (ranked according to *P*-value [smallest to largest]) (contained in Excel file – **Supplementary Information File 2**).

**Table S5:** DE genes showing **(a)** increased and **(b)** decreased expression at +6 wk post-infection relative to the -1 wk pre-infection control group (ranked according to *P*-value [smallest to largest]) (contained in Excel file – **Supplementary Information File 2**).

**Table S6:** DE genes showing **(a)** increased and **(b)** decreased expression at +10 wk post-infection relative to the -1 wk pre-infection control group (ranked according to *P*-value [smallest to largest]) (contained in Excel file – **Supplementary Information File 2**).

**Table S7:** DE genes showing **(a)** increased and **(b)** decreased expression at +12 wk post-infection relative to the -1 wk pre-infection control group (ranked according to *P*-value [smallest to largest]) (contained in Excel file – **Supplementary Information File 2**).

**Table** **S8:** Over-represented canonical pathways identified using IPA from DE genes detected between +2 wk post-infection and -1 wk pre-infection (ranked according to adjusted *P*-value [smallest to largest]). Statistically significant pathways are highlighted (light orange - B-H FDR adjusted *P*-value ≤ 0.05; dark orange - B-H FDR adjusted *P*-value ≤ 0.01) (contained in Excel file – **Supplementary Information File 3**).

**Table** **S9:** Over-represented canonical pathways identified using IPA from DE genes detected between +6 wk post-infection and -1 wk pre-infection (ranked according to adjusted *P*-value [smallest to largest]). Statistically significant pathways are highlighted (light orange - B-H FDR adjusted *P*-value ≤ 0.05; dark orange - B-H FDR adjusted *P*-value ≤ 0.01) (contained in Excel file – **Supplementary Information File 3**).

**Table** **S10:** Over-represented canonical pathways identified using IPA from DE genes detected between +10 wk post-infection and -1 wk pre-infection (ranked according to adjusted *P*-value [smallest to largest]). Statistically significant pathways are highlighted (light orange - B-H FDR adjusted *P*-value ≤ 0.05; dark orange - B-H FDR adjusted *P*-value ≤ 0.01) (contained in Excel file – **Supplementary Information File 3**).

**Table** **S11:** Over-represented canonical pathways identified using IPA from DE genes detected between +12 wk post-infection and -1 wk pre-infection (ranked according to adjusted *P*-value [smallest to largest]). Statistically significant pathways are highlighted (light orange - B-H FDR adjusted *P*-value ≤ 0.05; dark orange - B-H FDR adjusted *P*-value ≤ 0.01) (contained in Excel file – **Supplementary Information File 3**).

**Supplementary Table 12:** GO enrichment results for STEM model profile 40 generated from *STEM Analysis 1* (all detectable expressed genes). GO terms are ranked by *P*-value and GO terms with significant corrected *P*-values (≤ 0.05) are highlighted (contained in Excel file – **Supplementary Information File 4**).

**Supplementary Table 13:** GO enrichment results for STEM model profile 40 generated from *STEM Analysis 2* (union of all DE genes). GO terms are ranked by *P*-value and GO terms with significant corrected *P*-values (≤ 0.05) are highlighted (contained in Excel file – **Supplementary Information File 4**).

**Supplementary Table 14:** GO enrichment results for STEM model profile 23 generated from *STEM Analysis 1* (all detectable expressed genes). GO terms are ranked by *P*-value and GO terms with significant corrected *P*-values (≤ 0.05) are highlighted (contained in Excel file – **Supplementary Information File 4**).

**Supplementary Table 15:** GO enrichment results for STEM model profile 23 generated from *STEM Analysis 2* (union of all DE genes). GO terms are ranked by *P*-value and GO terms with significant corrected *P*-values (≤ 0.05) are highlighted (contained in Excel file – **Supplementary Information File 4**).

Supplementary Figures

**
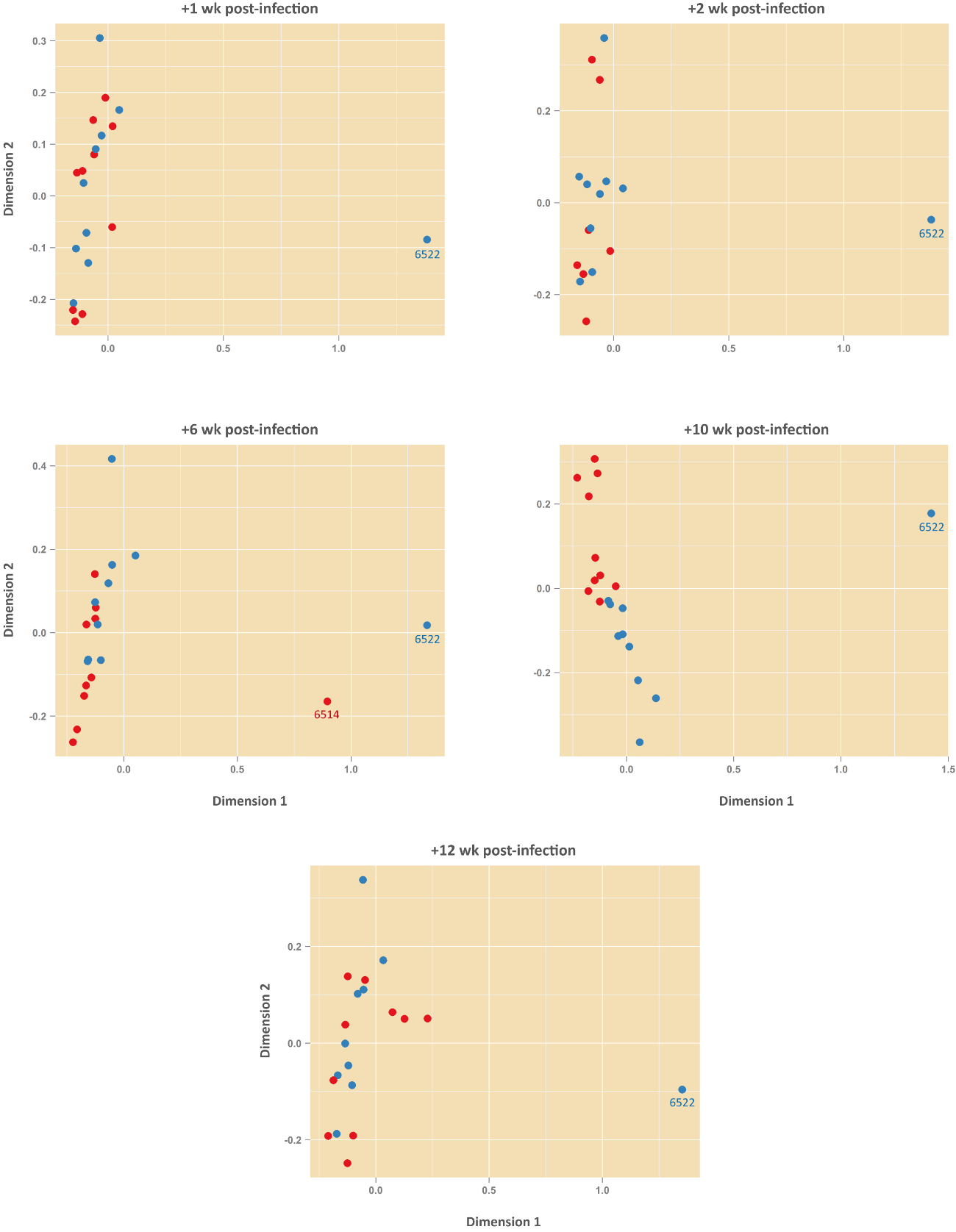
**

**Supplementary Figure 1: MDS plots generated from RNA-seq expression data (*n* = 10).** Results are shown for 12,406 filtered genes (57 animal samples) and individual plots show infected samples at each post-infection time point (red circles) and control samples at -1 wk pre-infection (blue circles). The outlier non-infected -1 wk control and infected +6 wk samples are labelled (sample 19, ID 6522 and sample 10, ID 6514).


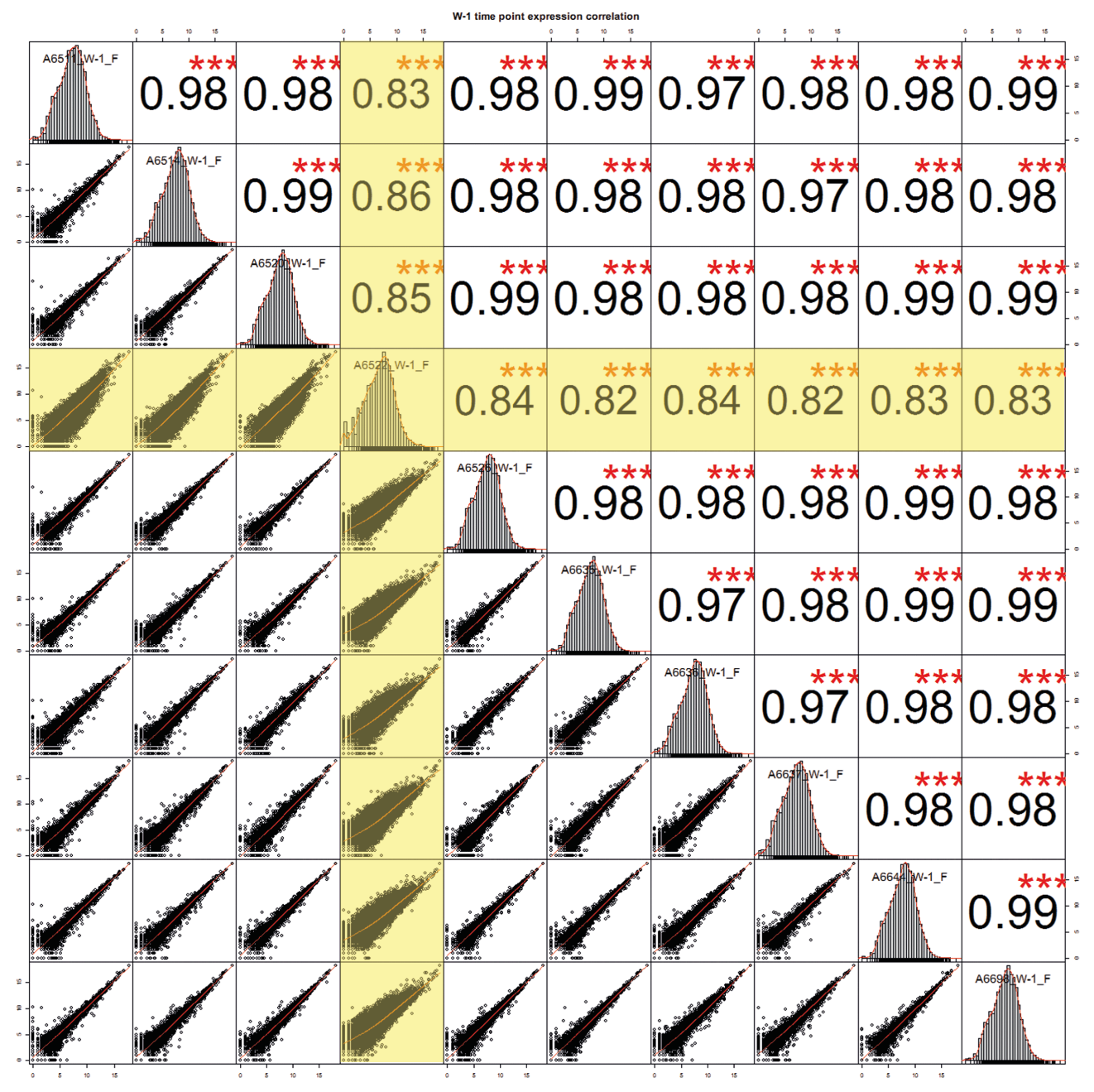


**Supplementary Figure 2: Spearman rank correlation plots and coefficients for RNA-seq data.** Results are shown for all pairwise comparisons of filtered RNA-seq gene expression data for ten control animal samples at -1 wk pre-infection. The results for the outlier (sample 19, ID 6522) are highlighted in yellow. Asterisks indicate significance of the correlations, which were all highly significant (*P* ≤ 0.001).


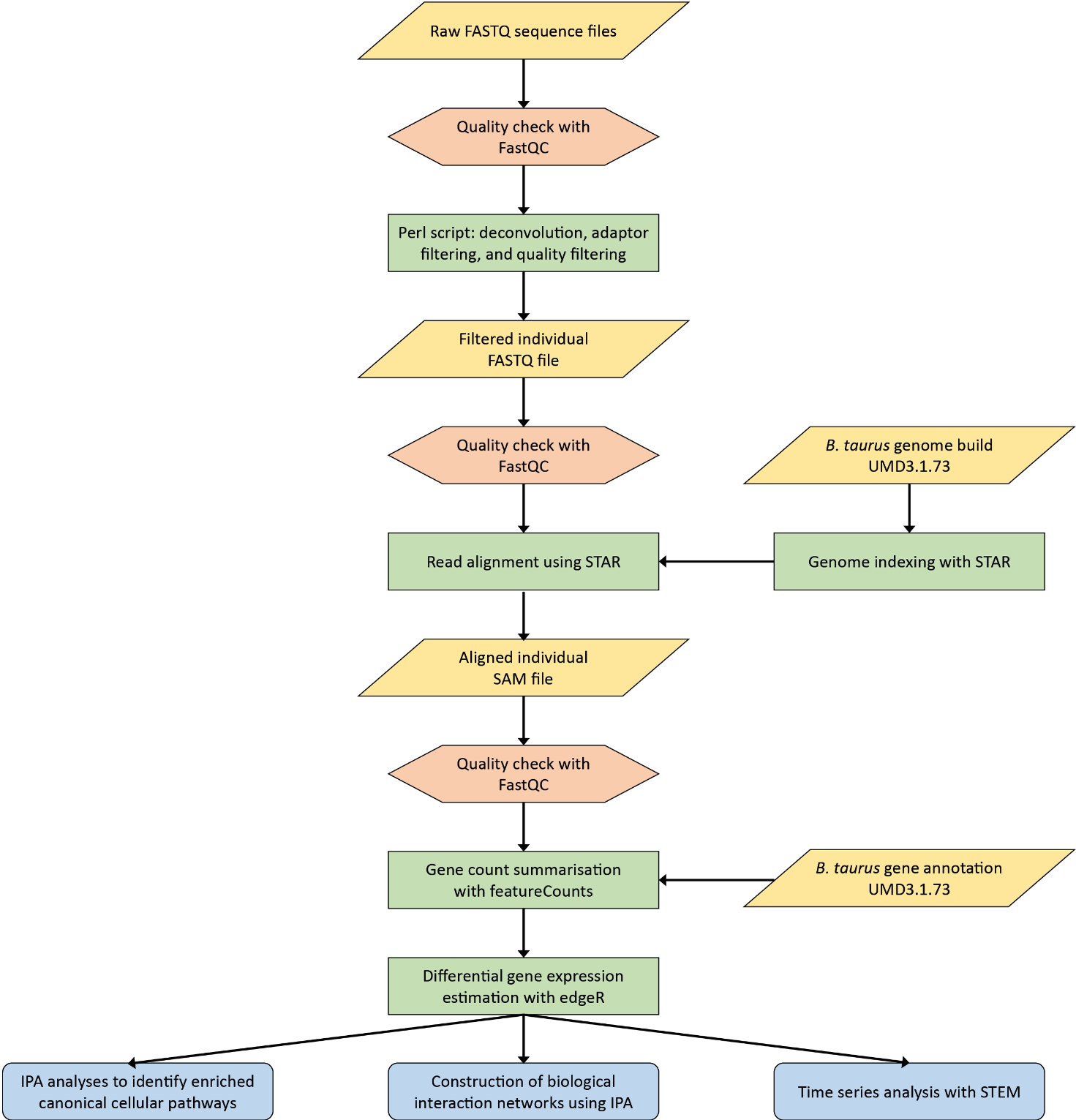


**Supplementary Figure 3: Bioinformatics workflow and computational pipeline used for the RNA‐seq differential gene expression and downstream analyses.**


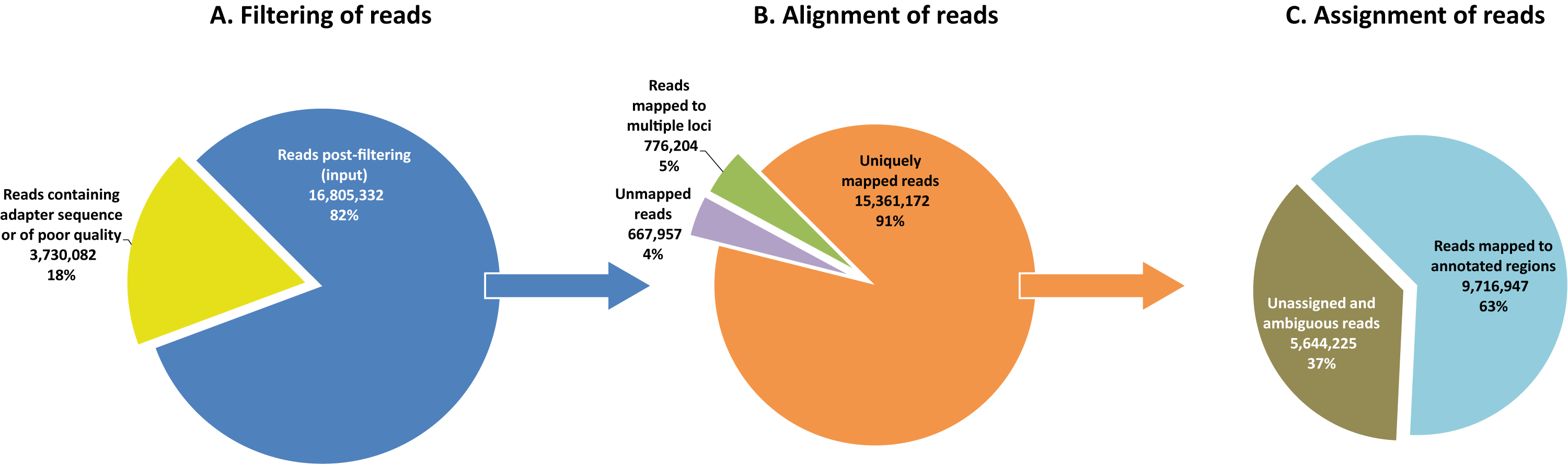


**Supplementary Figure 4: RNA-seq reads processing.** A) Pie chart with the mean number and percentage of sequence reads per libraries that were retained after deconvolution and filtering of reads; and those reads filtered out due to adapter sequence contamination or low sequence quality. B) Pie chart with the mean number and percentage of sequence reads per libraries that aligned to unique locations in the *B. taurus* reference genome, multiple locations in the *B. taurus* reference genome, and reads that did not align to any genomic locations. C) Pie chart with the mean number and percentage of uniquely mapped reads assigned to ambiguous gene features (i.e., reads that mapped to overlapping gene sequences), unidentified gene features (i.e., reads that map to the genome that have no gene annotation) and identified features (i.e., Ensembl genes).


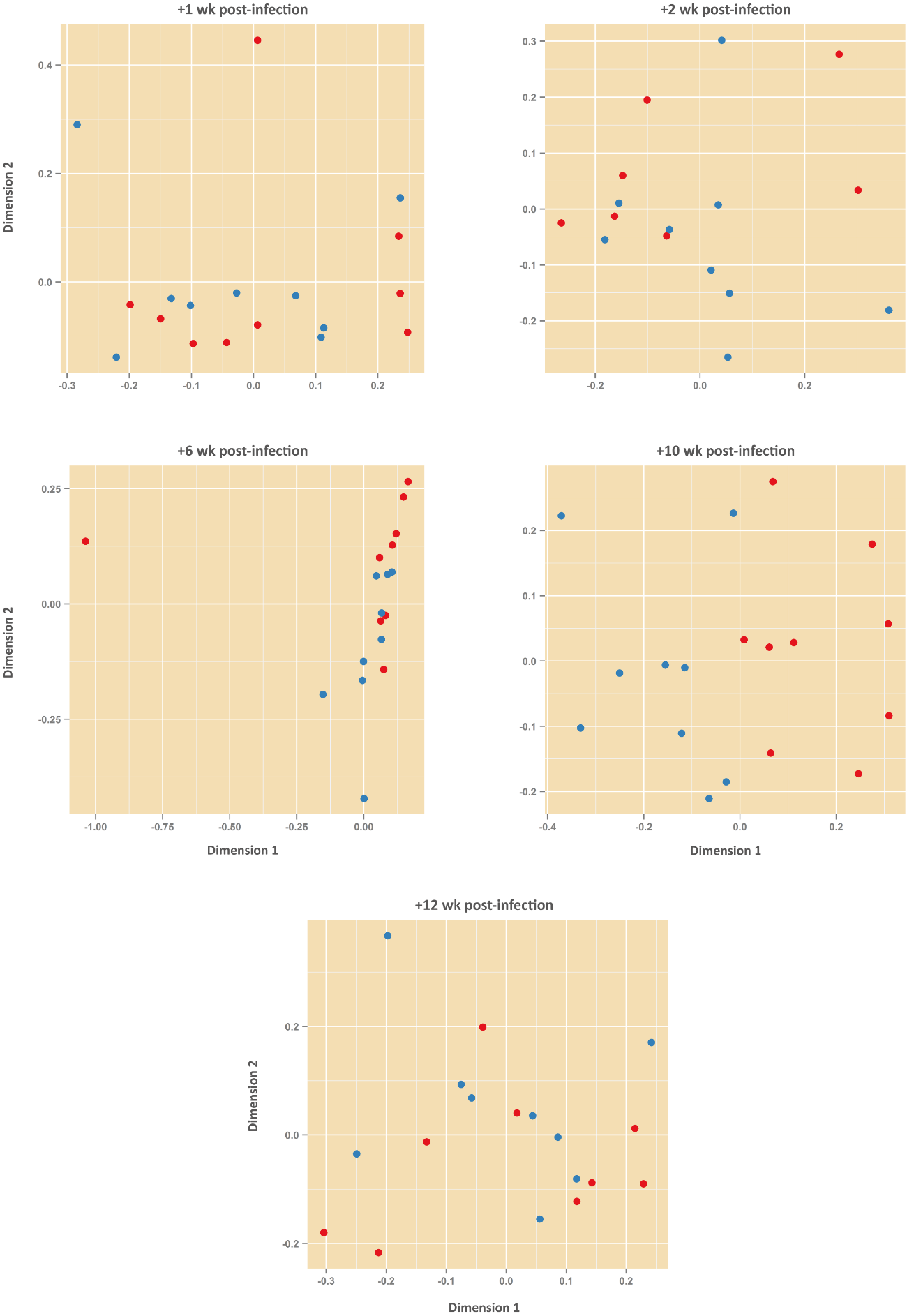


**Supplementary Figure 5: MDS plots generated from RNA-seq expression data (*n* = 9).** Results are shown for 12,406 filtered genes (52 animal samples). Individual plots show infected samples at each post-infection time point (red circles) and control samples at -1 wk pre-infection (blue circles). Animal ID 6522 was removed from all time points for this analysis and two additional technical dropouts were also excluded.


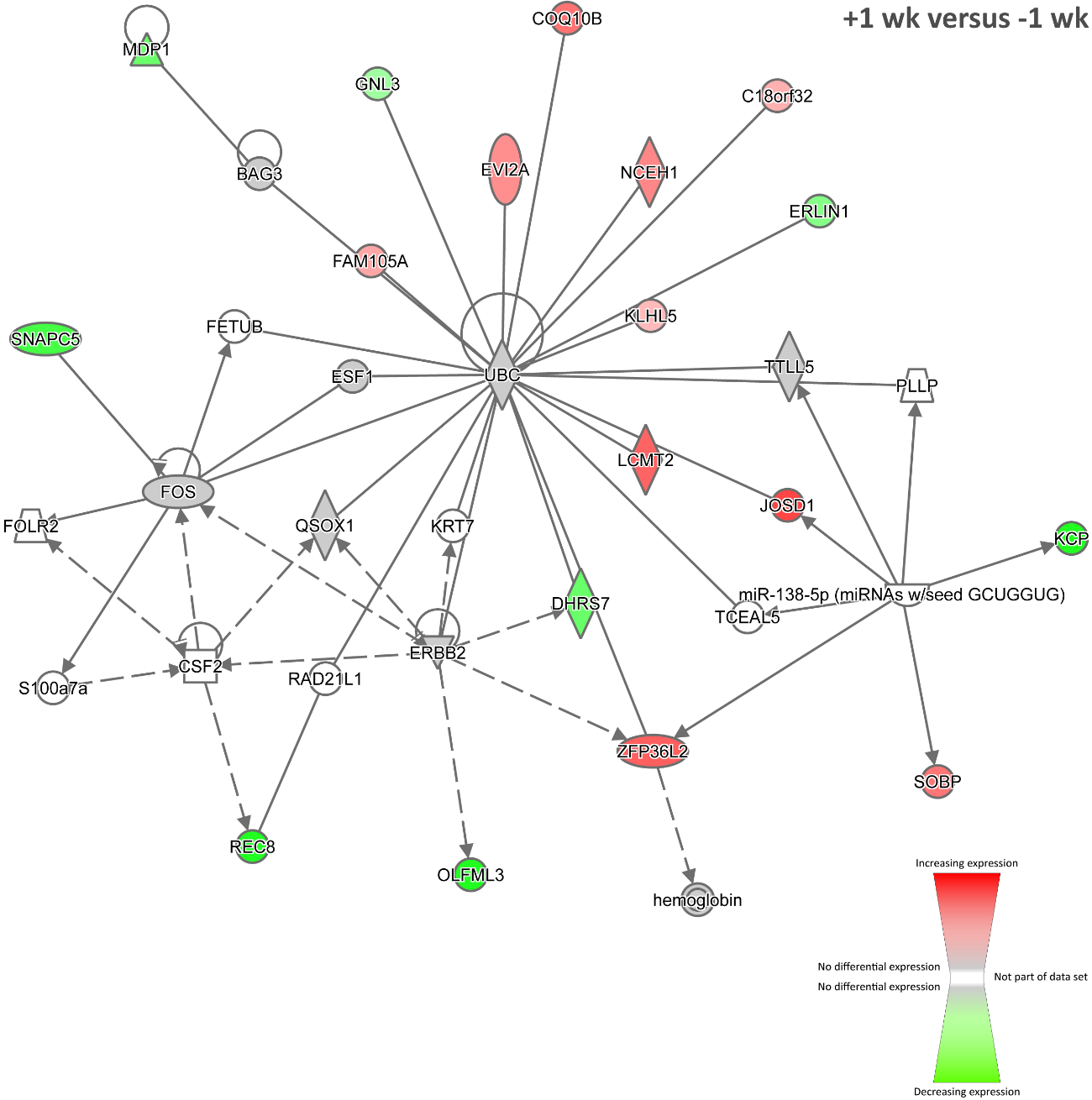


**Supplementary Figure 6: The top-ranked biological interaction network generated using IPA for +1 wk post-infection.** Differential gene expression is represented with a red-green colour scale. This network consisted of 18 focus molecules (IPA Network Score = 42) and the top IPA Disease and Function categories represented were Embryonic Development, Organismal Development, and Reproductive System Development and Function. A detailed legend for IPA biological interaction networks including a key for node shapes and edge classifications is available at the following link: <https://qiagen.secure.force.com/KnowledgeBase/articles/Basic_Technical_Q_A/Legend>.


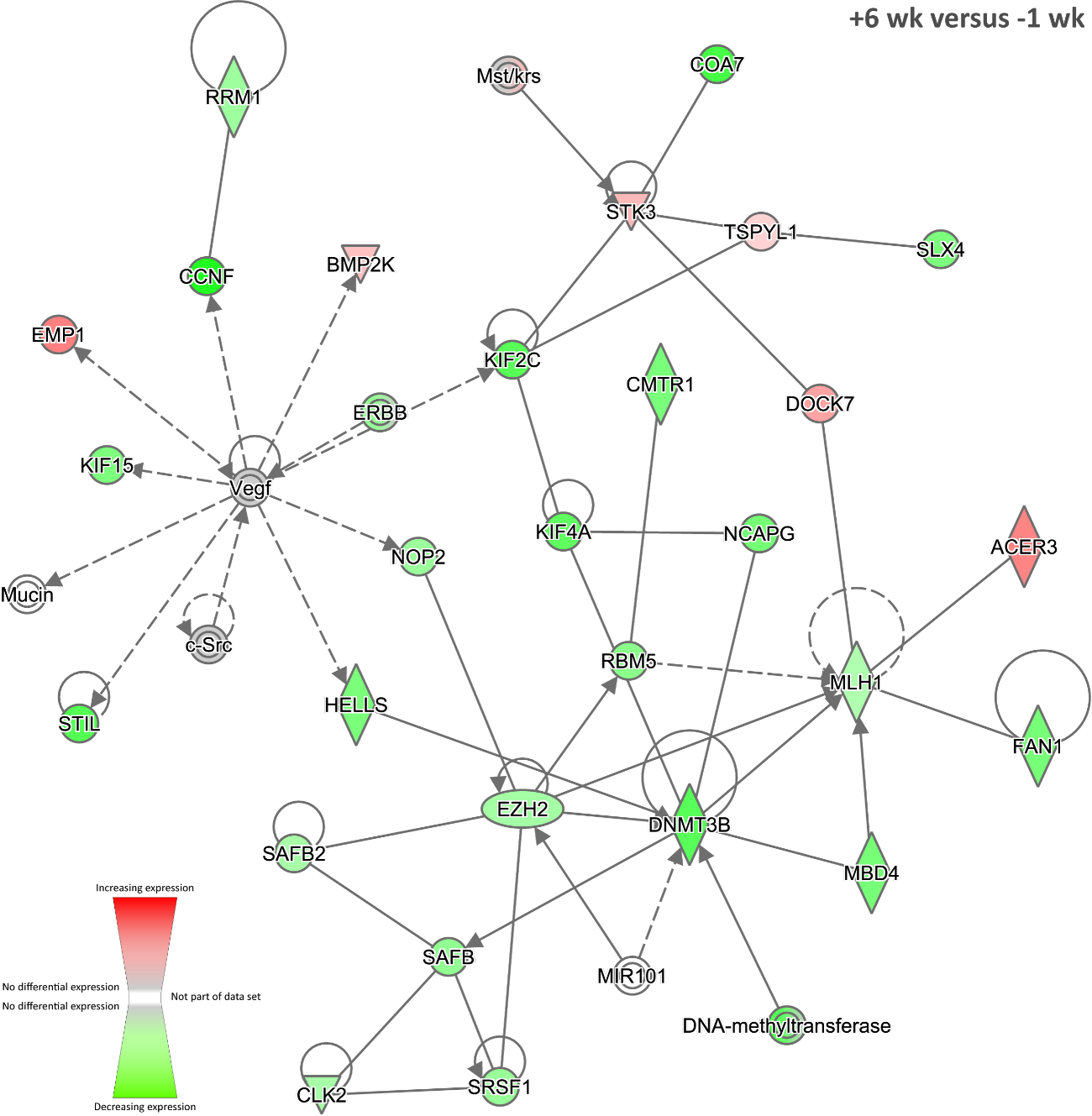


**Supplementary Figure 7: The top-ranked biological interaction network generated using IPA for +6 wk post-infection.** Differential gene expression is represented with a red-green colour scale. This network consisted of 28 focus molecules (IPA Network Score = 40) and the top IPA Disease and Function categories represented were DNA Replication, Recombination, and Repair, Gene Expression and Cell Cycle. A detailed legend for IPA biological interaction networks including a key for node shapes and edge classifications is available at the following link: <https://qiagen.secure.force.com/KnowledgeBase/articles/Basic_Technical_Q_A/Legend>.


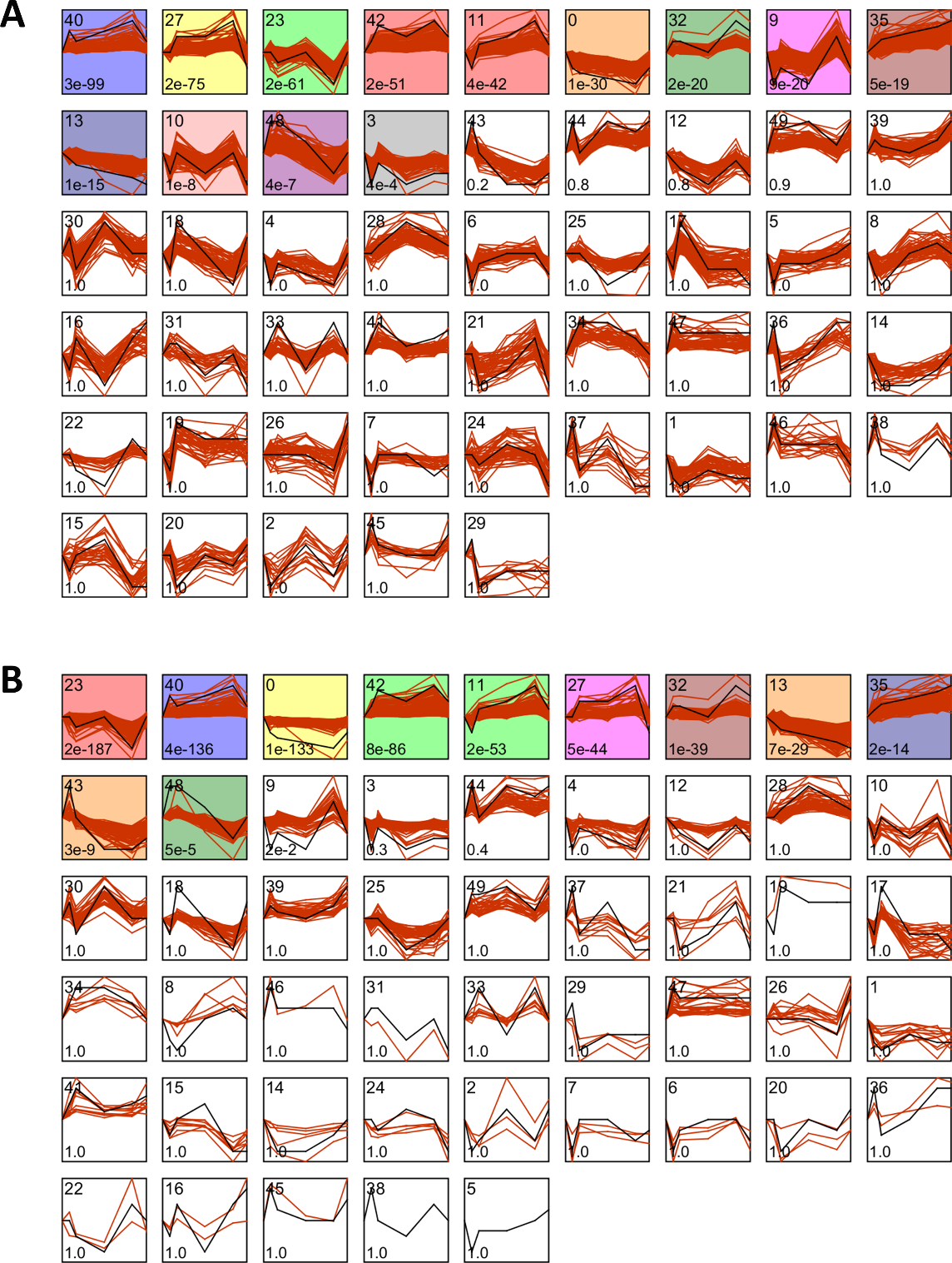


**Supplementary Figure 8: The top 50 time series model profiles for A) *STEM analysis 1* and B) *STEM analysis 2*.** The black lines show the model profiles generated using STEM for all filtered detectable genes (4,103 and 2,935 for *STEM analysis 1* and *2*, respectively) expressed across the infection time course (-1 wk, +1 wk, +2 wk, +6 wk, +10 wk and +12 wk). Model profiles are arrayed by statistical significance (shown in the bottom left corner of each profile). Statistically significant model profiles are coloured, and colour groups correspond to model profile clusters with similar expression profiles determined by correlation analysis. Model profile numbers (top left corner) correspond to order in which the STEM algorithm generates the profiles and does not relate to statistical significance of individual model profiles. The log_2_ expression values (relative to -1 wk pre-infection) for the genes associated with each model profile are overlaid at each time point and plotted in dark red. Note: the log_2_ expression values for all genes at time point -1 wk are equal to zero.


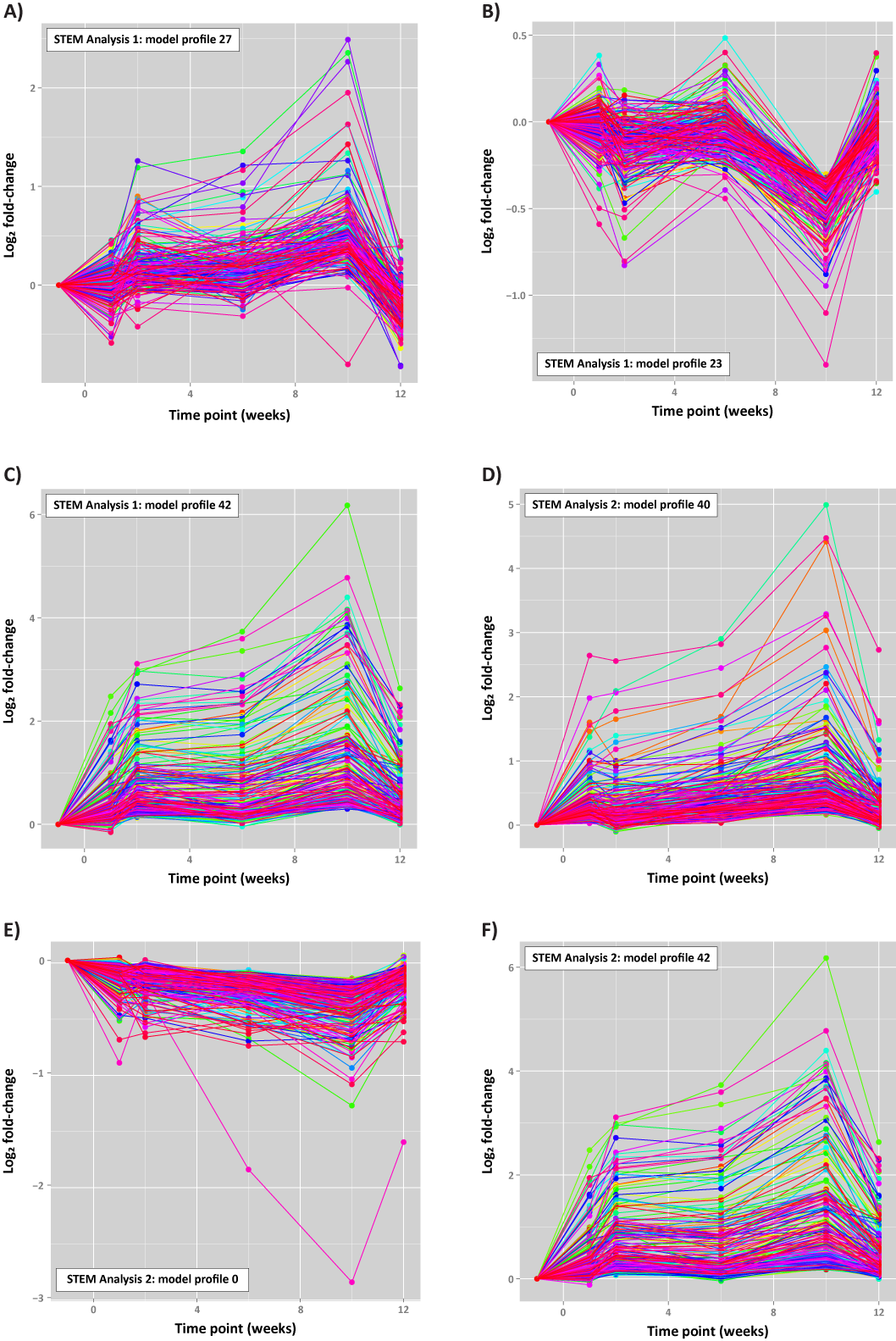


**Supplementary Figure 9: The four top-ranked profiles for *STEM analysis 1* and *STEM analysis 2*.** The 2^nd^- 3^rd^- and 4^th^-ranked model profiles for *STEM analysis 1* are shown in A, B, and C, respectively; and for *STEM analysis 2* in D, E, and F, respectively.
